# Multiple recombination events between endogenous retroviral elements and feline leukemia virus

**DOI:** 10.1128/jvi.01400-23

**Published:** 2024-01-19

**Authors:** Minh Ha Ngo, Loai AbuEed, Junna Kawasaki, Naoki Oishi, Didik Pramono, Tohru Kimura, Masashi Sakurai, Kenji Watanabe, Yoichi Mizukami, Haruyo Ochi, Yukari Anai, Yuka Odahara, Daigo Umehara, Maki Kawamura, Shinya Watanabe, Ariko Miyake, Kazuo Nishigaki

**Affiliations:** 1 Laboratory of Molecular Immunology and Infectious Disease, Joint Graduate School of Veterinary Medicine, Yamaguchi University, Yoshida, Yamaguchi, Japan; 2 Faculty of Science and Engineering, Waseda University, Okubo, Shinjuku-ku, Tokyo, Japan; 3 Oishi Animal Clinic, Oshu-shi, Iwate, Japan; 4 Joint Graduate School of Veterinary Medicine, Yamaguchi University, Yoshida, Yamaguchi, Japan; 5 Laboratory of Veterinary Pathology, Joint Faculty of Veterinary Medicine, Yamaguchi University, Yoshida, Yamaguchi, Japan; 6 Institute of Gene Research, Science Research Center, Yamaguchi University, Minami-kogushi, Ube, Japan; 7 Life Science Division, Advanced Technology Institute, Yamaguchi University, Yoshida, Yamaguchi, Japan; Icahn School of Medicine at Mount Sinai, New York, New York, USA

**Keywords:** feline leukemia virus, endogenous retroviruses, genetic recombination, transmission, pathogenesis, domestic cats

## Abstract

Feline leukemia virus (FeLV) is an exogenous retrovirus that causes malignant hematopoietic disorders in domestic cats, and its virulence may be closely associated with viral sequences. FeLV is classified into several subgroups, including A, B, C, D, E, and T, based on viral receptor interference properties or receptor usage. However, the transmission manner and disease specificity of the recombinant viruses FeLV-D and FeLV-B remain unclear. The aim of this study was to understand recombination events between exogenous and endogenous retroviruses within a host and elucidate the emergence and transmission of recombinant viruses. We observed multiple recombination events involving endogenous retroviruses (ERVs) in FeLV from a family of domestic cats kept in one house; two of these cats (ON-T and ON-C) presented with lymphoma and leukemia, respectively. Clonal integration of FeLV-D was observed in the ON-T case, suggesting an association with FeLV-D pathogenesis. Notably, the receptor usage of FeLV-B observed in ON-T was mediated by feline Pit1 and feline Pit2, whereas only feline Pit1 was used in ON-C. Furthermore, XR-FeLV, a recombinant FeLV containing an unrelated sequence referred to the X-region, which is homologous to a portion of the 5′-leader sequence of *Felis catus* endogenous gammaretrovirus 4 (FcERV-gamma4), was isolated. Genetic analysis suggested that most recombinant viruses occurred *de novo*; however, the possibility of FeLV-B transmission was also recognized in the family. This study demonstrated the occurrence of multiple recombination events between exogenous and endogenous retroviruses in domestic cats, highlighting the contribution of ERVs to pathogenic recombinant viruses.

**IMPORTANCE:**

Feline leukemia virus subgroup A (FeLV-A) is primarily transmitted among cats. During viral transmission, genetic changes in the viral genome lead to the emergence of novel FeLV subgroups or variants with altered virulence. We isolated three FeLV subgroups (A, B, and D) and XR-FeLV from two cats and identified multiple recombination events in feline endogenous retroviruses (ERVs), such as enFeLV, ERV-DC, and FcERV-gamma4, which are present in the cat genome. This study highlights the pathogenic contribution of ERVs in the emergence of FeLV-B, FeLV-D, and XR-FeLV in a feline population.

## INTRODUCTION

Endogenous retroviruses (ERVs) are germline remnants of ancestral infections caused by exogenous retroviruses and are present in all vertebrate genomes (1). ERVs comprise a substantial proportion of the mammalian genome and are transmitted in a Mendelian manner (2). When retroviruses are inserted into the host genome, they achieve increased copy numbers through the repeated reinfection of germline cells (3). Eventually, ERVs become inherited components (4). Most ERVs lack the ability to code for infectious viruses due to mutations in the viral genome (5), and replication-competent ERVs rarely exist (6, 7). ERVs are known to influence the pathogenesis of exogenous retroviruses (exRVs). After infection with exRVs, recombination may occur between hereditary ERVs and exRVs, altering the genetic makeup of exRVs and resulting in the generation of new infectious agents that cause severe disease (7).

Feline leukemia virus (FeLV) belongs to the family *Retroviridae* and genus *Gammaretrovirus*. The viral genome contains two long terminal repeats (LTRs) and three main retroviral genes: *gag*, *pol*, and *env* (8). FeLV infections have been reported in domestic cats worldwide, including in Europe, the United States, South America, Japan, Australia, and New Zealand (9–14). FeLV has the highest mortality rate among all of the major feline viruses affecting domestic cats, including feline immunodeficiency virus and feline coronavirus (15). The clinical signs of FeLV infection vary, with approximately 60% of infections resulting in aborted or shortened infections; however, the remainder may progress to high levels of thymic lymphoma, multicentric lymphoma, myelodysplastic syndrome, acute myeloid leukemia, aplastic anemia, and immunodeficiency (8). Predicting the emergence of a specific disease phenotype in FeLV-infected cats is challenging due to the variability in natural FeLV infection outcomes.

FeLV is classified into several subgroups, including A, B, C, D, T, and TG35-2 (classified as FeLV-E), based on viral receptor interference properties or receptor usage (16–29). Viral interference tests have been used to identify FeLV subgroups that were later found to be associated with subgroup-specific phenotypes of the virus (30). Feline thiamine transporter 1 (feTHTR1) is a receptor for FeLV-A (21), phosphate transporters (Pit1 and Pit2) serve as receptors for FeLV-B (22, 23), feline leukemia virus subgroup C receptor-related proteins (FLVCRs) are receptors for FeLV-C (24–26), feline cupper transporter 1 (feCTR1) is a receptor for FeLV-D (28), Pit1 with FeLIX (co-factor) is a receptor for FeLV-T (29), and feline reduced folate carrier (RFC) (feRFC) is a receptor for FeLV-E (27). FeLV-A is transmitted among cats through grooming, biting, blood, feces, and mother’s milk (31–35). FeLV-B has been identified, along with FeLV-A, in isolates from approximately 40% of natural infections and is detected in a high percentage of cats with lymphoma (9, 35). It has been reported that FeLV-B was generated through the recombination of the endogenous FeLV envelope gene (*env*) with FeLV-A, as demonstrated *in vitro* (36). However, a comparison of the viral recombination site in naturally infected cats revealed that the position is not the same as that in any of the recombinants (9), suggesting that FeLV-B typically appears independently, rather than being transmitted. FeLV-B is unable to establish cell-free infection (37) and appears to require helper FeLV-A viruses for successful infection (38). However, there have been reports of cases of interspecies transmission of FeLV-B in wild cats (39) or under certain conditions, such as in multi-cat households (40).

ERV-DCs are endogenous gammaretroviruses of domestic cats (19, 41, 42); they are classified into genotypes I, II, and III according to their phylogenetic relationships, and the receptor of genotype I was identified as CTR1 (28, 43). Due to the presence of some genotypes and entry mechanisms of ERV-DCs, ERV-DC has been inserted into the feline genome several times in the past. Some ERV-DCs have intact open reading frames that encode *gag*, *pol*, and envelope (*env*) genes; therefore, they are replication-competent in cultured cells and can infect cells. Furthermore, FeLV-D was generated through the recombination of genotype I ERV-DCs in the *env* region (19) and exhibits distinct tropism, compared with other FeLV subgroups. Importantly, ERV-DCs retain their ability to affect their hosts through their potential viral activity and their contribution to the emergence of recombinant viruses. In addition, one ERV-DC recombined with Baboon endogenous retrovirus (BaEV) and generated a distinct feline retrovirus, RD-114 (19, 41, 42). This past recombination event suggests interspecies retroviral transmission between cats and primates (19, 41, 42) and that the interactions between exogenous retroviruses and ERVs have contributed to long-term retroviral diversification and evolution. However, the transmission manner and disease specificity of FeLV-D and FeLV-B remain unclear.

In our previous study, we identified the X-region containing FeLV (XR-FeLV), a recombinant FeLV containing an unrelated sequence referred to as the X-region (44). This region is homologous to a portion of the 5′-leader sequence and the *gag* gene of *Felis catus* endogenous gammaretrovirus 4 (FcERV-gamma4), which is present in *Felis catus*. The frequency of recombinant FeLV carrying the X-region was 6.4% and was associated with disease progression, such as lymphoma, leukemia, and renal failure. However, the function of the X-region remains unknown (44).

Although FeLV is well documented, little is known about the recombination events that occur in natural infections. Understanding the molecular underpinnings of this process allows us to gain a deeper understanding of the FeLV disease, as well as important details regarding endogenous-exogenous retroviral interactions. Here, we describe recombination events between exogenous and endogenous retroviruses within a host based on viral isolation and the pathobiological analyses of infected cats. This research also focuses on the emergence and transmission of recombinant viruses, shedding light on the possible emergence of novel recombinant viruses. This study demonstrates the occurrence of multiple recombination events between exogenous and endogenous retroviruses in domestic cats, highlighting the contribution of ERVs to pathogenic viruses.

## RESULTS

### Status of FeLV and Feline immunodeficiency virus infection in a family of cats

**Fig 1:**
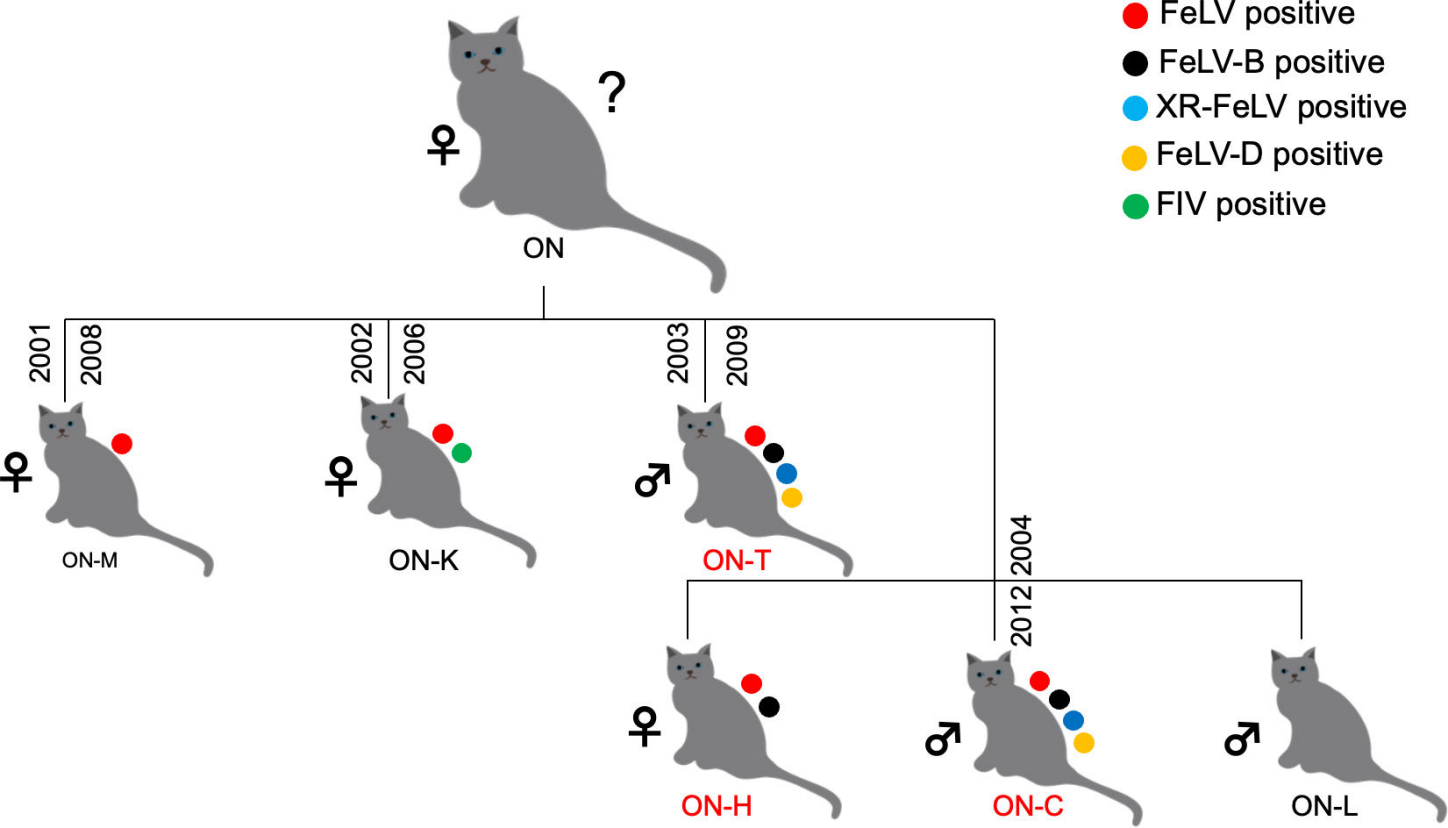
History of the domestic cat family. In the family with the prefix ON, a mother cat gave birth to four separate litters, as indicated in the figure. The red circles indicate FeLV-antigen positive cats, and the green circle indicates the FIV-antibody positive cat detected using a commercial kit. FeLV-B-positive cats are represented by black circles determined based on the cloning of FeLV-B *env* genes, FeLV-D-positive cats are represented by yellow circles, and XR-FeLV-positive cats are represented by blue circles, as detected by PCR analysis. The branch is marked with the year of birth and death. The dates of death for ON-H and ON-L were not available. Samples from ON-T, ON-H, and ON-C were used for virus isolation and gene cloning of FeLV; the FeLV-positive animals are marked with red dots. Animals that were further characterized in the present study (ON-T, ON-H, ON-C) are highlighted in red.

In the family tree prefixed with ON, five (ON-M, ON-K, ON-T, ON-C, and ON-H) of the six cats (excluding mother cat, ON) were infected with FeLV, based on FeLV-antigen testing using a commercial kit, and two (ON-C and ON-T) were infected with FeLV-D, based on PCR analysis performed in a previous study (19). In the present study, feline immunodeficiency virus (FIV), FeLV-B, and XR-FeLV infection, along with an evaluation of FeLV-D infection, was tested in the family (Fig. 1). Two of the six cats (excluding mother cat) were positive for FeLV-D and XR-FeLV infection, as ascertained via PCR (Fig. S1). Three cats (ON-T, ON-C, and ON-H) were infected with FeLV-B, as ascertained based on the cloning of the *env* gene for FeLV-B (Table 1) (described subsequently). Only one sample (ON-K) was found to be positive for anti-FIV antibodies using a commercial kit. Samples of ON-M and ON-K for genetic analysis were not available. In the present study, viruses were newly isolated from ON-T and ON-C using different samples and analyzed in detail, and these included the previous viral isolate obtained from the spleen of ON-T [293T/ON-T/#0 cells (19)] (Table 1); this isolate has not been well-characterized. Furthermore, genetic analyses of FeLVs from ON-T, ON-C, and ON-H were performed.

**TABLE 1:** Viral isolation and list of FeLV Env and FeLV provirus clones from ON-T, ON-C, and ON-H^*e*^

Cat ID	Source^*a*^	Cell lines^*b*^	FeLV-A	FeLV-B	FeLV-D	XR-FeLV
ON-T	Spleen	293T/ON-T/#0^*c*^	None	ON-T_clone_9 (LC765251) ON-T_clone_11 (LC765250)	FeLV-D/ re-T (AB673432)^*c*^ ON-T_clone_1.1 (LC765256) ON-T_clone_1.2 (LC765257) ON-T_clone_1.3 (LC765258)	None
	PBMCs	293T/ON-T/#3^*d*^	ON-T_clone_3.2 (LC765236) ON-T_clone_3.4 (LC765237) FeLV-A/ONT_clone_10 (LC765228)	ON-T_clone_3.6 (LC765248) ON-T_clone_3.14 (LC765249)	ON-T_clone_3.1 (LC765254) ON-T_clone_3.2 (LC765255)	FeLV-A/ON-T_ provirus_clone_10 (LC765228)
	Spleen		ON-T_clone_1.1 (LC765238) ON-T_clone_1.2 (LC765239)	ON-T_clone_2 (LC765246) ON-T_clone_14 (LC765247)	FeLV-D/ON-T (AB673426)^*c*^	N.D.
ON-C	Plasma	AH927/ON-C/#9	ON-C_clone_9.2 (LC765232) ON-C_clone_9.4 (LC765233) FeLV-A/ON-C_ provirus_clone_30 (LC765227)	ON-C_clone_9.1 (LC765243) ON-C_clone_9.14 (LC765244)	ON-C_clone_9.21 (LC765252) ON-C_clone_9.22 (LC765253) FeLV-D/ON-C_provirus_clone_ 20 (LC765229)	FeLV-D/ON-C_ provirus_clone_20 (LC765229)
	Blood		ON-C _clone_3 (LC765230) ON-C _clone_4 (LC765231)	ON-C_clone_1 (LC765240) ON-C_clone_13 (LC765242) ON-C_clone_14 (LC765241)	FeLV-D/ON-C (AB673429)^*c*^ ON-C_clone_11 ON-C_clone_13 ON-C_clone_14.2 ON-C_clone_15.1 ON-C_clone_15.2	N.D.
ON-H	Blood		ON-H_clone_1 (LC765234) ON-H_clone_2 (LC765235)	ON-H_clone_16 (LC765245)	None	None

^*a*^ Indicates the materials used for viral isolation or FeLV PCR cloning.

^*b*^ Indicates the established cell lines for viral isolation.

^*c*^ Indicates previously reported cell lines and viral clones (19).

^*d*^ Peripheral blood mononuclear cells (PBMCs) from ON-T were co-cultured with feline AH927 cells, and the supernatant was inoculated into human HEK293T cells.

^*e*^ The clones are listed with the gene accession numbers. PBMCs, peripheral blood mononuclear cells; XR-FeLV, X-region containing FeLV; N.D., not determined.

### Clinical signs and histopathological features

In the ON-C case, clinical signs included decreased activity and appetite; however, no signs of respiratory disease were observed. Blood analysis of ON-C indicated a high white blood cell count, anemia, and a low platelet count (93,200 /µL white blood cells, 4,200,000 /µL red blood cells, 30.7% hematocrit, and 138,000 /µL platelets), leading to a leukemia diagnosis. Simultaneously, the ON-T case developed conjunctivitis, ear dermatitis, and otitis externa. ON-T was found to have severe anemia (hematocrit level of approximately 10%) and was diagnosed with lymphoma of the thoracic cavity. Necropsy of ON-T revealed rheum in the eyes and mucosal pallor in the gums (Fig. S2A and B). In addition, a mass was found within the thoracic cavity and in a section of the heart base (Fig. S2C and D).

Histopathological analyses of the heart mass and tissues (liver, kidney, and spleen) from ON-T indicated infiltration with lymphoid cells (Fig. S2E through L), which were classified as mature based on their morphology. Additionally, atypical erythrocytes, atypical erythroblasts, atypical megakaryocytes, and macrophages were observed in the heart mass and tissues (Fig. S2E through L).

### Clonality analysis of FeLV-D provirus integration in ON-T

**Fig 2:**
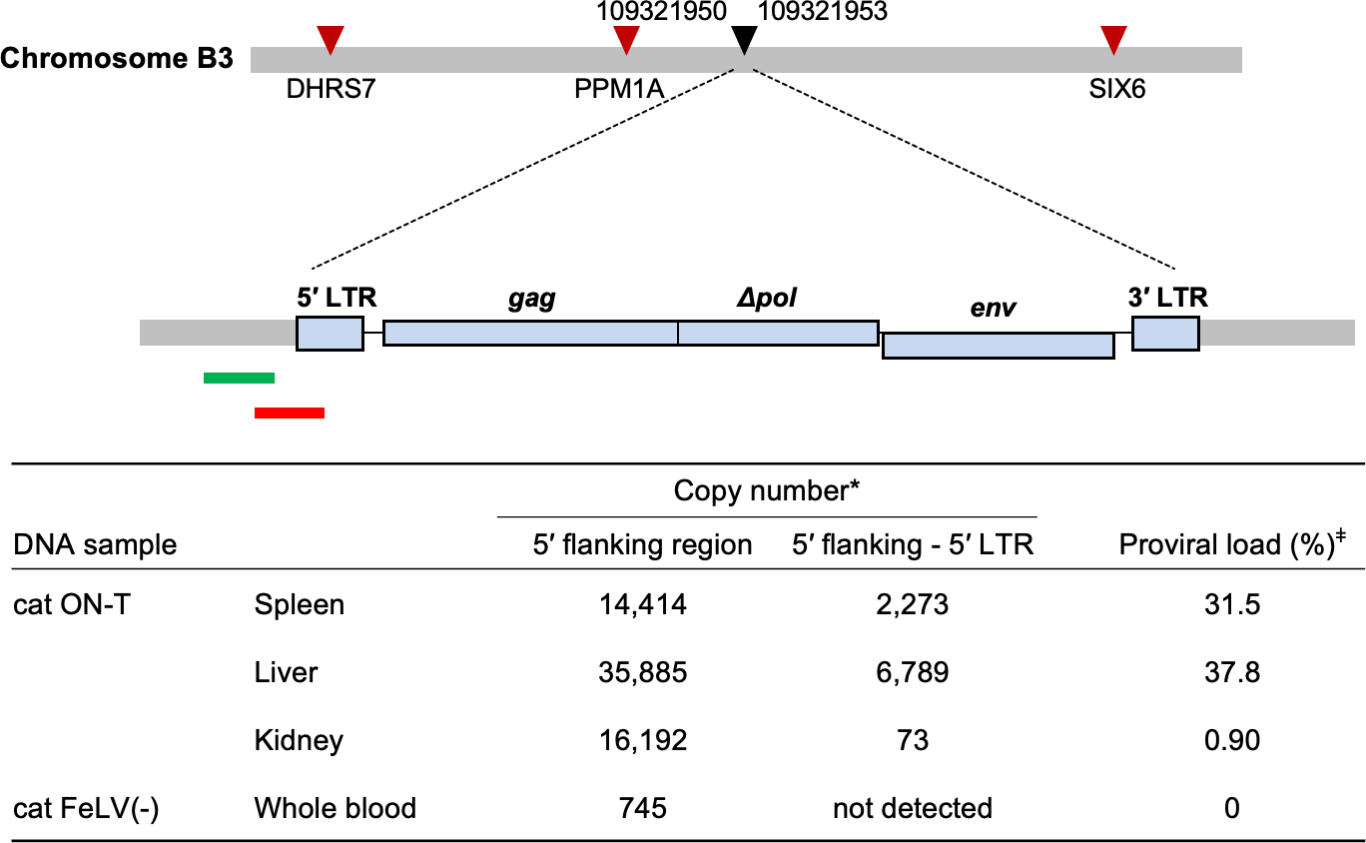
Clonal integration of FeLV-D in ON-T. Schematics of FeLV-D proviral genome highlighting the functional features: the 5' long terminal repeat, *gag*, *pol*, *env,* and 3' LTR. The green (5' flanking region) and red (5' flanking–5' LTR junction) bars indicate the area targeted by quantitative PCR. The FeLV-D/ON-T ((AB673426) integrated genomic position was determined using BLAT from the University of California Santa Cruz genome browser (https://genome.ucsc.edu/cgi-bin/hgBlat). The black triangle indicates the integration site of the FeLV-D provirus. The red triangles indicate the location of the genes for protein phosphatase PPM1A, sine oculis-related homeobox 6 (Six6), and dehydrogenase/reductase 7 (DHRS7). *Copy number was per 2 µL of DNA sample. ^ǂ^Proviral load (%) = [Copy number of “5'flanking–5'LTR”/(Copy number of “5'flanking region”/2)] × 100.

We aimed to determine the presence of clonal integration of the FeLV-D provirus. The FeLV-D/ON-T provirus integrated into the genome at positions 109321950 to 109321953 on chromosome B3, which was previously molecularly cloned (Fig. 2) (19). After analyzing the viral integration site using the genome database (https://genome.ucsc.edu/), protein phosphatase PPM1A, sine oculis-related homeobox 6 (Six6), and dehydrogenase/reductase 7 (DHRS7) were expected to be present in the vicinity of the FeLV-D integration site (Fig. 2). Using this molecular clone, we conducted quantitative real-time PCR to assess the clonal integration of the FeLV-D provirus. A high copy number of the FeLV-D provirus was detected in the spleen and liver, with proviral loads of 31.5% and 37.8%, respectively (Fig. 2). A low copy number of FeLV-D was detected in the kidney, with a proviral load of 0.9%. These results indicated that there was clonality in the integration of FeLV-D, suggesting that FeLV-D might be involved in tumorigenesis (see Discussion).

### FeLV-A, FeLV-B, and FeLV-D isolation from cats

Previously, we isolated FeLV-D from the homogenized spleens of ON-T co-cultured with human HEK293T cells (termed 293T/ON-T/#0 cells, Table 1) (19). In this study, we attempted to isolate FeLVs from ON-C and ON-T and analyzed them simultaneously. Samples from other cats in the ON family were not available as materials for cell cultures. Peripheral Blood Mononuclear Cells (PBMC) from ON-T were co-cultured with feline AH927 cells, and the supernatant was inoculated into HEK293T cells. Both AH927 and 293T cells are susceptible to FeLV infection. We also directly added the plasma from ON-C into AH927 cells. After culturing the cells for several weeks, chromosomal DNA was extracted and the FeLV *gag* gene was detected by PCR, confirming the isolation of FeLV from ON-T and ON-C. The resulting cultured cells were termed 293T/ON-T/#0, 293T/ON-T/#3, and AH927/ON-C/#9, respectively (Table 1). We also attempted to isolate FeLV from the plasma of ON-C via addition into HEK293T cells but were unsuccessful in isolating FeLVs in these cells (293T/ON-C/#8).

Next, we conducted an interference assay to evaluate the presence of FeLV subgroups in the cultured cells (Table 2). The results indicated that FeLV-B and FeLV-D Env-pseudotyped viruses were blocked in 293T/ON-T/#0 cells; FeLV-A, FeLV-B, and FeLV-D Env-pseudotyped viruses were blocked in 293T/ON-T/#3 cells; and FeLV-A, FeLV-B, and FeLV-D Env-pseudotyped viruses were blocked in AH927/ON-C/#9 cells. The FeLV-C and FeLV-E TG35-2 Env-pseudotyped viruses were not blocked in any of the cell lines. These results suggest that 293T/ON-T/#0 cells contained FeLV-B and FeLV-D, 293T/ON-T/#3 cells contained FeLV-A, FeLV-B, and FeLV-D, and AH927/ON-C/#9 cells contained FeLV-A, FeLV-B, and FeLV-D.

**TABLE 2:** Viral interference assay^*a*^^,^^*d*^^,^^*e*^

	Pseudotyped viruses^*c*^					Mock
	FeLV-A	FeLV-B	FeLV-C	FeLV-D	FeLV-E	
	/Clone 33	/GA	/Sarma	/ON-T	/TG35-2	
Viral receptor	THTR1	Pit	FLVCR	CTR1	RFC	
293T cells	+	++	++	++^*b*^	++	–
293T/ON-T/ #0 cells	+	–	++	–	++	–
293T/ON-T /#3 cells	–	–	++	+^*b*^	++	–
AH927 cells	++	++	++	+	++	–
AH927/ON-C /#9 cells	–	–	++	–	++	–

^*a*^ –, infection titer of 0; +, 1 to 10^3^ IU/mL; ++, 10^3^ to 10^5^ IU/mL. IU, infectious units.

^*b*^ indicates a statistically significant difference between these cell lines (*P* < 0.05).

^*c*^ The virus strains used in the assay are shown.

^*d*^ THTR1, thiamine transporter 1; Pit, phosphate transporter; FLVCR, feline leukemia virus subgroup C receptor-related protein; CTR1, cupper transporter 1; RFC, reduced folate carrier.

^*e*^ These data represent the results of three independent experiments.

### Genetic analysis of FeLV-A *env* genes from ON-T, ON-C, and ON-H

**Fig 3:**
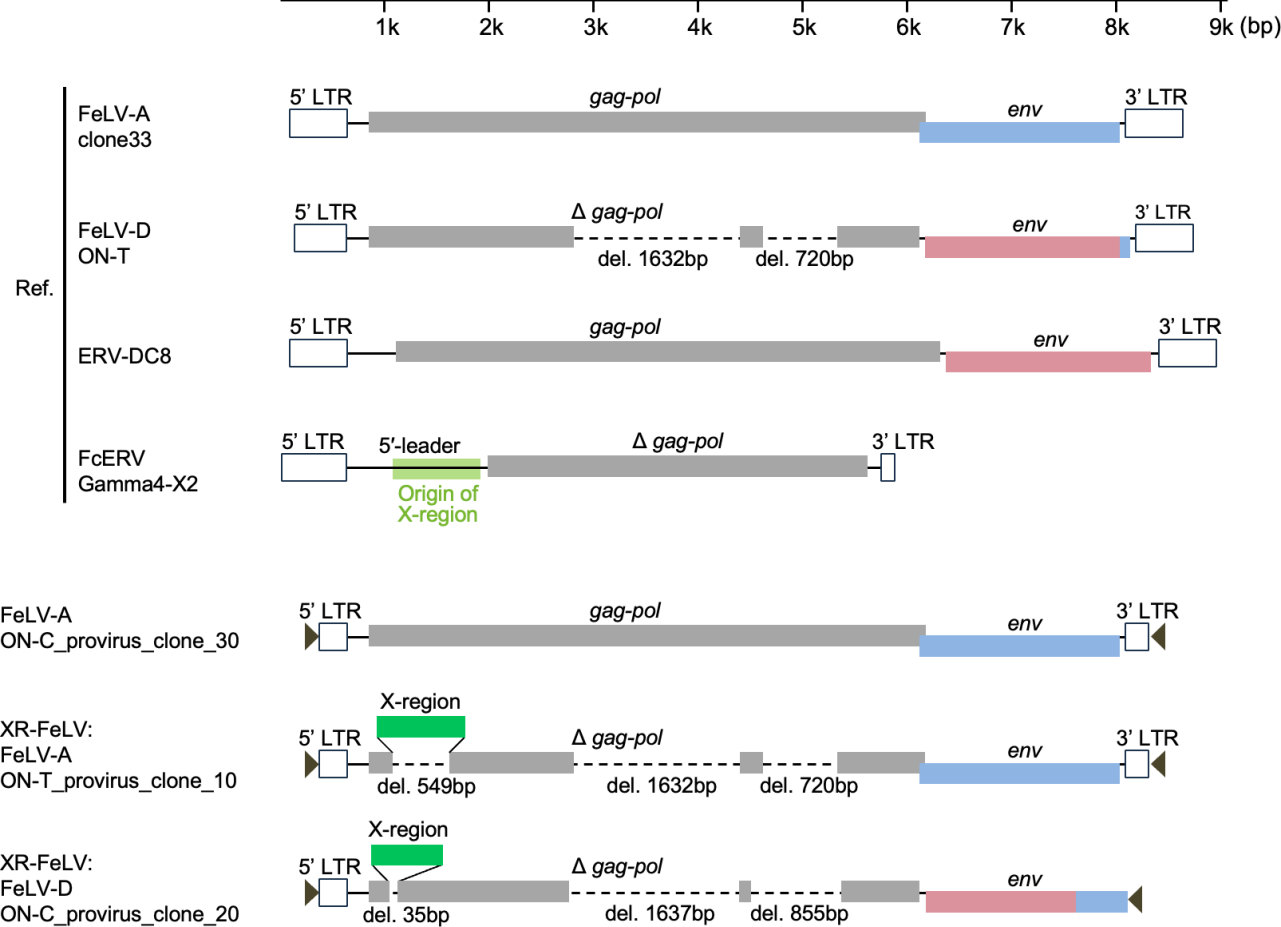
Schematic structure of recombinant FeLV proviruses isolated from cell lines. The FeLV-A/ON-C_provirus_clone_30 and FeLV-D/ON-C_provirus_clone_20 were isolated from the AH927/ON-C/#9 cell line, and the FeLV-A/ON-T_provirus_clone 10 was isolated from the 293T/ON-T/#3 cell line. FeLV-A/clone33, FeLV-D/ON-T, FcERV-gamma4-X2, and ERV-DC8 were referenced. The genes are illustrated as 5′-leader, *gag-pol*, *env*, or Δ*gag-pol*, along with the 5ʹ- and 3ʹ-LTRs. Δ*gag-pol* contains a deletion of the gene. The gray boxes indicate predicted *gag-pol* or Δ*gag-pol*. The blue boxes indicate FeLV-A *env* or the origin of FeLV-A *env*. The pink boxes indicate ERV-DC *env* or the origin of ERV-DC *env*. The dashed lines indicate gene deletions, and the sizes of the deletions are indicated. The green boxes indicate the X-region in XR-FeLV, and the origin of the X-region is also marked in light green on FeERV-Gamma4-X2. The bar indicates the scale; del, deletion; Ref, reference. Arrowheads indicate PCR primers for provirus cloning.

**Fig 4:**
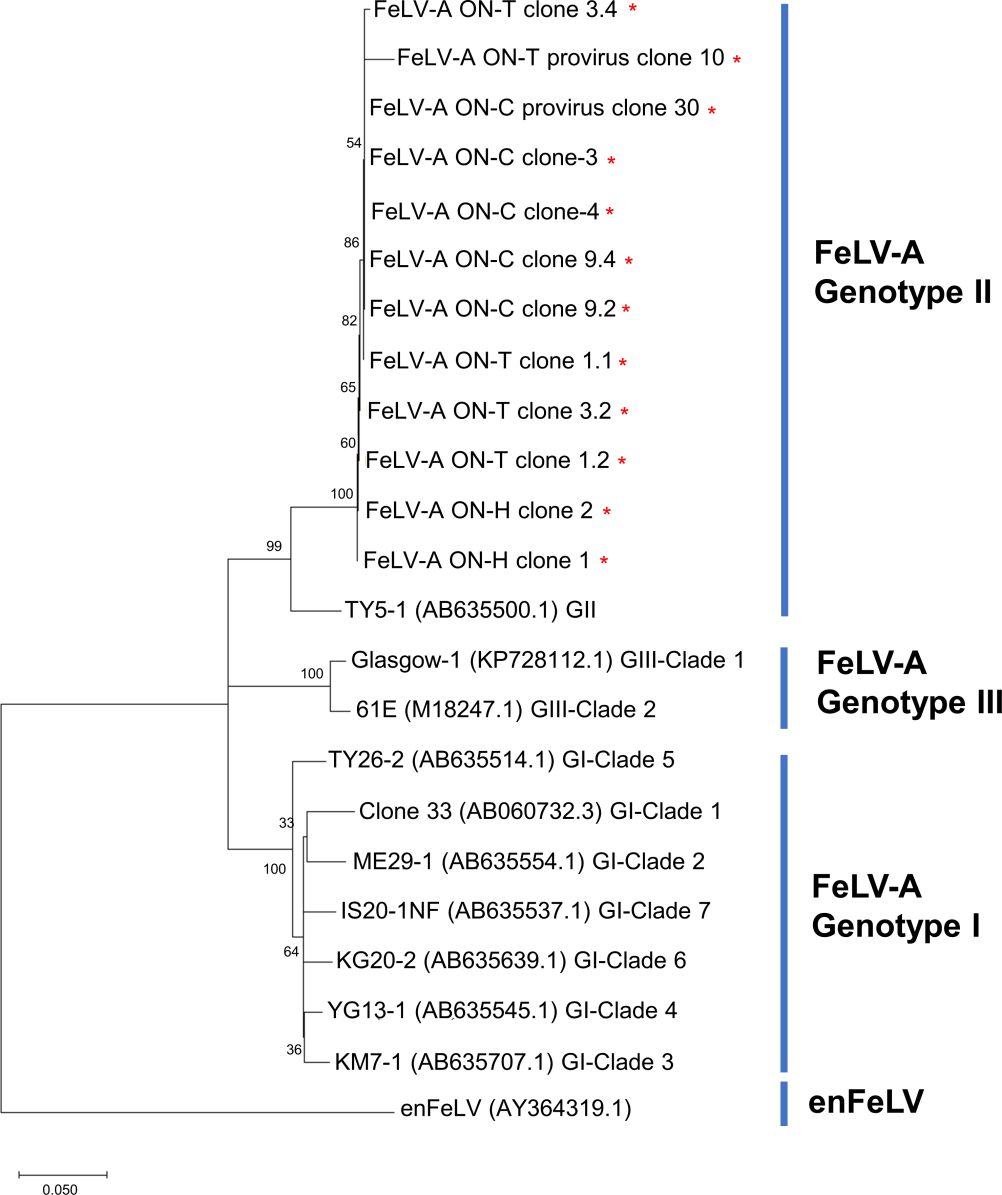
Phylogenetic analysis of FeLV-A. Maximum likelihood phylogenetic trees were constructed based on the General Time Reversible model using the nucleotide sequence for the FeLV-A *env* region. The bootstrap values (%) with 1,000 replicates are indicated on the branches. The tree is drawn to scale, with branch lengths measured by the number of substitutions per site. The FeLV-A genotype is indicated as Genotype I (GI), Genotype II (GII), and Genotype III (GIII). The clade classification of GI and GIII is shown. The red asterisks indicate the FeLV-A clones isolated in this study.

Next, we performed PCR analysis to clone and sequence the FeLV *env* genes and nearly full-length proviruses from viral isolates and tissues obtained from three cats (ON-T, ON-C, and ON-H) kept in one house (Table 1; Fig. 3). Phylogenetic analysis using FeLV-A *env* genes suggested three classifications, namely, Genotype I, II, and III (Fig. 4) (9). All FeLV-A *env* genes from ON-T, ON-C, and ON-H were classified into Genotype II, and low genetic diversity of FeLV-A was observed (Fig. 4). These results suggest that FeLV-A transmission was likely to have occurred within the family.

### Existence of FeLV-B transmission within the household

**Fig 5:**
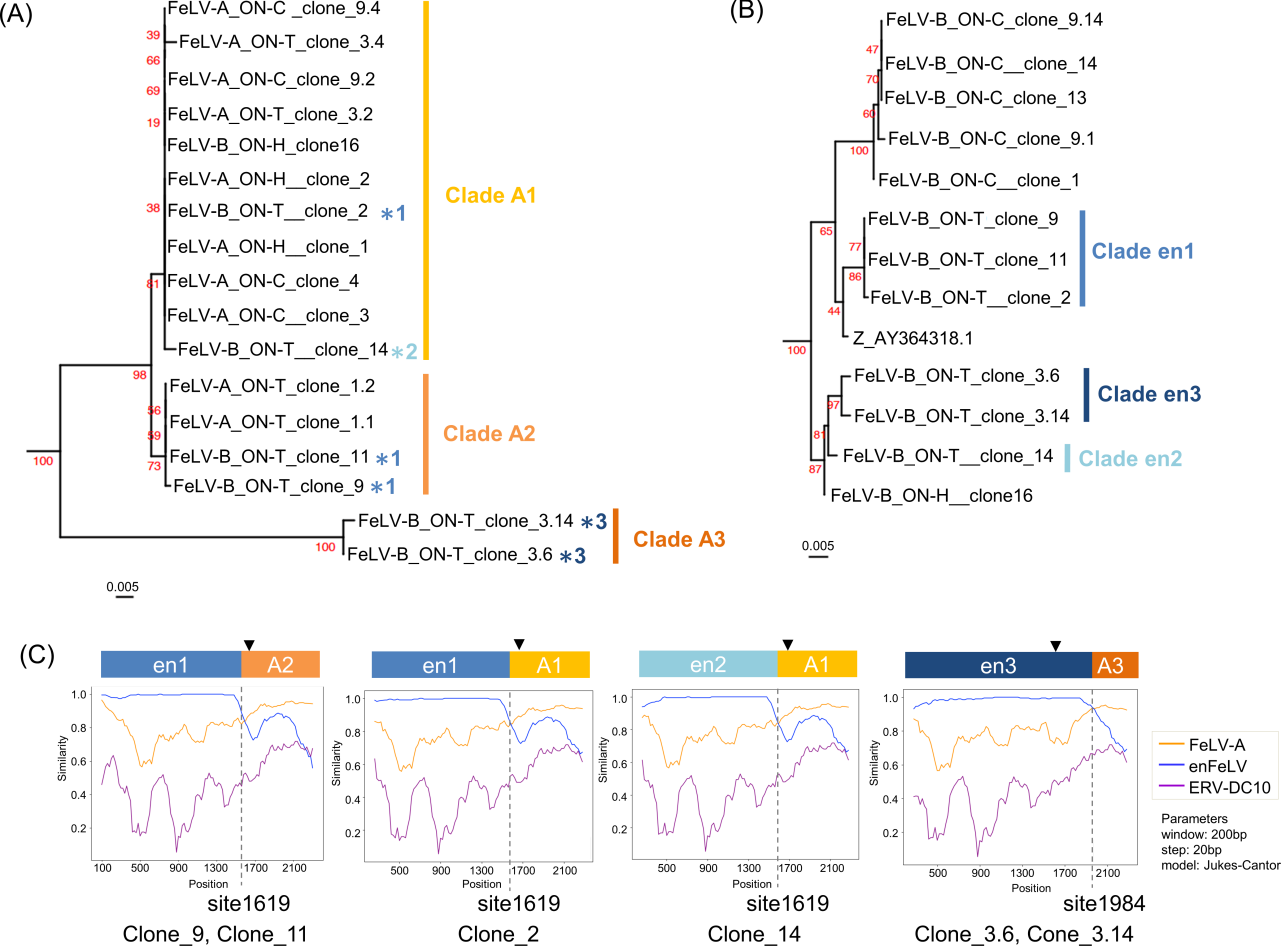
Phylogenetic and recombination junction analyses of FeLV-B *env* genes. (**A**) Maximum likelihood phylogenetic trees were constructed using nucleotide sequences of the FeLV-A *env* region and (**B**) enFeLV *env* region. The (**B**) tree contains an enFeLV sequence indicated by Z (Accession No. AY364318.1). The blue asterisks [light blue (asteristik2), blue (asteristik1), and dark blue (asteristik3) colors] in (**A**) correspond to clades en1–3 in (**B**). (**C**) Recombination junction of FeLV-B identified from ON-T. Simplot ++ analysis was used to identify enFeLV- and FeLV-A-derived (light orange, orange, and dark orange colors) regions in the FeLV-B *env* sequence (details in Fig. S3). The filled colors and indexes correspond to clades classified via phylogenetic analysis in (**A**) and (**B**). Surface unit (SU) and transmembrane (TM) junctions are marked with arrowheads in Fig. 6C. The plots in panel C do not include the start and stop sites in the *env* gene. The nucleotide position of the analyzed genome region is displayed on the *x*-axis, and the percentage of nucleotide identities between the query sequence and the reference strain is shown on the *y*-axis. Recombination site is indicated by a dotted line. Filled triangle indicates a cleave site of viral envelope (surface unit and transmembrane).

We performed two analyses to investigate the possibility of FeLV-B transmission within the household. First, we checked for concordance or discordance in the recombination junctions of FeLV-B detected in the ON family cats (Fig. S3). Second, we performed phylogenetic analyses using the enFeLV and FeLV-A regions from which FeLV-B had originated (Fig. 5). Simplot analysis identified three recombination junctions in 12 FeLV-B sequences from the ON family cats (Fig. S3). The first recombination junction (around site 1192) was identified only in FeLV-B from ON-C, suggesting that FeLV-B emerged *de novo* in ON-C. The second junction (around site 1984) was detected in clone 3.6 and clone 3.14 of ON-T. The third junction (around site 1619) was shared among the viruses identified in ON-T (clones 2, 9, 11, and 14) and ON-H (clone 16), suggesting inter-individual FeLV-B transmission between ON-T and ON-H.

To further confirm whether FeLV-B was transmitted between these cats, phylogenetic analyses were conducted using the FeLV-A and enFeLV regions (Fig. 5A and B). As the result, ON-H FeLV-B clone 16 and ON-T FeLV-B clone 14, which shared the same recombinant junction, were classified into the same clade in the phylogenetic tree using the FeLV-A region (clade A1 in Fig. 5). Furthermore, they were classified into the same lineage/clade in the phylogenetic tree using the enFeLV region (lineage/clade containing clade en2 and en3 in Fig. 5B).

These results suggest that the FeLV-B variant was transmitted between ON-T and ON-H.

### Diversification of FeLV-B in ON family cats

In addition to the possibility of inter-individual transmission of FeLV-B, this study also indicated that FeLV-B variants were diversified due to multiple recombination events in ON-T. Integrated analysis of the recombinant junctions and phylogenetic analysis identified four FeLV-B variants in ON-T (Fig. 5C). In addition, simplot analysis revealed two recombinant junctions (around sites 1619 and 1984) in FeLV-B ON-T. Notably, phylogenetic analysis demonstrated that the enFeLV regions of FeLV-B ON-T could be classified into three clades (clade en1–en3 in Fig. 5B). In contrast, the FeLV-A regions were classified into three clades (clades A1–A3 in Fig. 5A) but did not correspond to the classification based on the enFeLV region. These results suggest that diverse FeLV-B variants were generated in the ON family cats due to multiple recombination events.

### Determination of distinct FeLV-B strains based on receptor usage in ON-T and ON-C isolates

As FeLV-B was present in the viruses isolated from ON-T and ON-C, differences in infection based on viral receptor usage were assessed. By evaluating viral infection using *Mus dunni* tail fibroblast (MDTF) cells, MDTF cells expressing feline Pit1 (MDTF-fePit1), and MDTF cells expressing feline Pit2 (MDTF-fePit2), we found that the FeLV-B strain from ON-C (AH927/ON-C/#9 cells) infected MDTF-fePit1 cells, whereas the FeLV-B strains from ON-T (293T/ON-T/#3) infected both MDTF-fePit1 and MDTF-fePit2 cells (Table 3). These findings suggest that there are two distinct FeLV-B strains with different receptor usages.

**TABLE 3:** Viral infection assay^*c*^

Cell line	Supernatant		Mock
	293T/ON-T/ #3^*a*^	AH927/ON-C/ #9^*b*^	
293T	(1.57 ± 0.18) × 10^5^	(3.64 ± 1.27) × 10^4^	0
AH927	(2.76 ± 0.37) × 10^4^	(1.74 ± 0.35) × 10^4^	0
MDTF	0	0	0
MDTF/fePit1	(1.55 ± 0.19) × 10^5^	(1.80 ± 0.56) × 10^4^	0
MDTF/fePit2	(1.81 ± 0.39) × 10^4^	0	0

^*a*^ Supernatant harvested from 293Lac cells infected with supernatant of 293T/ON-T/ #3 cells was used for infection.

^*b*^ Supernatant harvested from AH927Lac cells infected with supernatant of AH927/ON-C/ #9 cells was used for infection. Titers (IU/mL) were determined by X-Gal staining. IU, infectious units.

^*c*^ The data represent the means and standard deviations of 2–4 independent experiments.

It has been reported that the arginine residue at position 73 in the variable region A (VRA) of the Env protein is important for envelope binding to feline Pit2 but not feline Pit1 (23). Our sequencing results indicated that FeLV-B *env* genes from ON-T and ON-H had an arginine at position 73, whereas FeLV-B *env* genes from ON-C had a glutamine (Fig. S4). These results might explain the differences in the receptor usages of FeLV-B strains; it was assumed that FeLV-B from ON-T and ON-H utilized both feline Pit1 and fePit2, while ON-C utilized only feline Pit1 as the receptor.

### Genetic analysis of FeLV-D *env* genes from ON-T and ON-C

**Fig 6:**
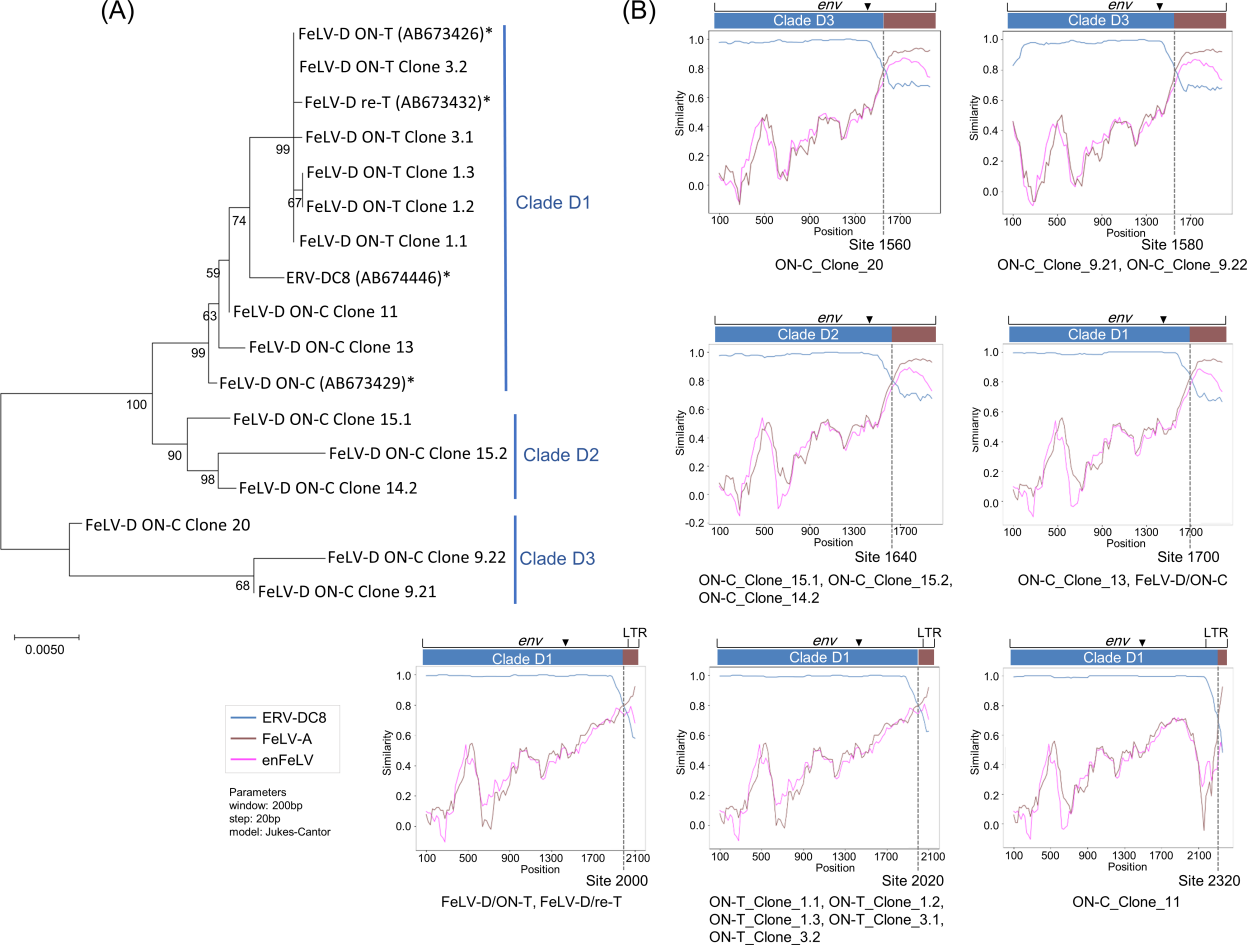
Phylogenetic and recombination junction analyses of FeLV-D *env* genes. (**A**) Maximum-likelihood phylogenetic tree using the nucleotide sequence of the ERV-DC region. The asterisks represent the previously reported sequence, FeLV-D/re-T, FeLV-D/ON-T, and ERV-DC8. FeLV-D was classified into Clade D1, Clade D2, and Clade D3. (**B**) Recombination junction of FeLV-D identified from ON family cats. Simplot ++ analysis was used to identify the ERV-DC8 (blue) and FeLV-A (brown) regions in the FeLV-D *env* sequence (details in Fig. S5). The nucleotide position of the analyzed genome region is displayed on the *x*-axis, and the percentage of nucleotide identities between the query sequence and the reference strain is shown on the *y*-axis. The lines indicate enFeLV (purple), FeLV-A (brown), and ERV-DC8 (blue). Recombination site is indicated by a dotted line. Filled triangle indicates a cleave site of viral envelope (surface unit and transmembrane).

Next, we cloned FeLV-D *env* genes and the provirus (clone 20) from blood (ON-C) and cell lines (293T/ON-T/#0, and 293T/ON-T/#3, and AH927/ON-C/#9) using PCR. To investigate whether FeLV-D was transmitted within the ON family, recombination junction and phylogenetic analyses were performed using *env* sequences identified in this study, and previously reported sequences [FeLV-D/re-T, FeLV-D/ON-T, and FeLV-D/ON-C (19)] (Fig. 6A and B; Fig. S5). Simplot analysis indicated the presence of 7 recombination junctions in 16 FeLV-D *env* regions. Five different recombination junctions (site 1560, 1580, 1640, 1700, and 2320) were identified in ON-C: FeLV-D with recombination sites 1560 and 1580 from viral isolates in AH927/ON-C/#9 cells, and FeLV-D with recombination sites 1640, 1700, and 2320 from molecular clones from a blood sample. Two recombination junctions were identified in ON-T: FeLV-D with recombination sites 2000 and 2020 from spleen and viral isolates in 293T/ON-T/#0 and 293T/ON-T/#3 cells. The different recombination events suggested that FeLV-D was not transmitted among the cats from the same household (ON-T and ON-C). Notably, most of the FeLV-Ds had recombination junctions within the *env* gene, but only FeLV-D ON-C_Clone 11 had a recombination junction upstream of U3 in the LTR.

We performed phylogenetic analysis of FeLV-D using the ERV-DC region, but we were unable to use the FeLV-A *env* regions due to its short sequence. These ERV-DC-derived sequences were classified into three clades, clade D1, clade D2, and clade D3. Clade D1 contained FeLV-D from ON-C and ON-T; however, the recombinant junctions were different.

These results support the hypothesis that FeLV-D emerged *de novo*.

### Isolation and characterization of XR-FeLV from the ON family cats

**Fig 7:**
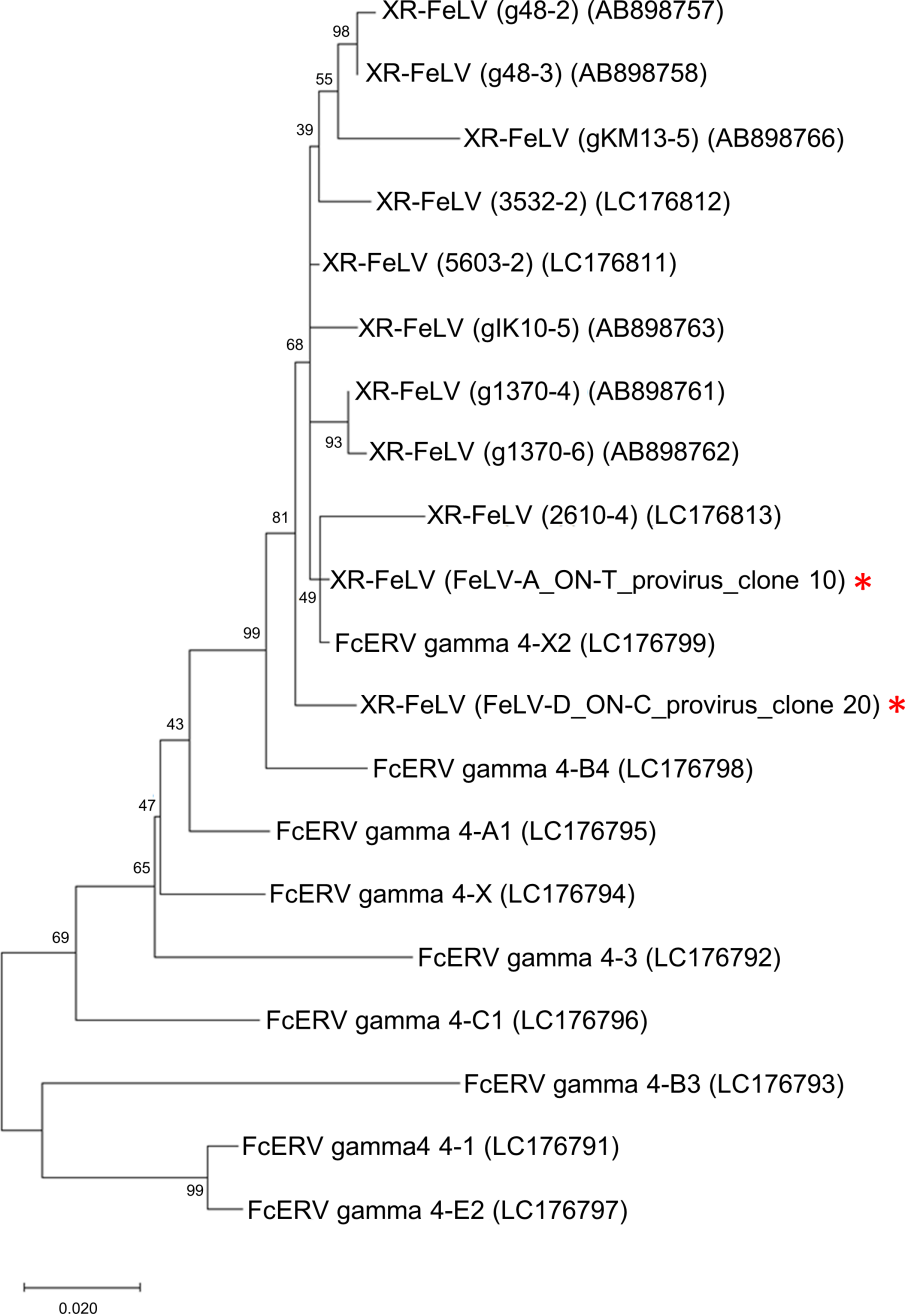
Phylogenetic analysis of X-region from XR-FeLV. Maximum Likelihood phylogenetic tree based on the General Time Reversible model. The percentage of trees in which the associated taxa clustered together is shown next to the branches. The tree is drawn to scale, with branch lengths measured by the number of substitutions per site. This analysis involved 20 nucleotide sequences. The asterisks marked in red indicate the XR-FeLV clones identified in this study.

The X-region containing FeLV (XR-FeLV) is a recombinant virus that has been recombined by the 5′-leader sequence and the *gag* gene of *F. catus* endogenous gammaretrovirus (FcERV-gamma4) (44). PCR was used to determine whether the ON family contained X-region-carrying recombinant FeLVs. We identified XR-FeLVs in blood samples from both ON-T and ON-C (Fig. S1). These viruses were also detected in cultured cells (293T/ON-T/#3 and AH927/ON-C/#9) but not in 293T/ON-T/#0 cells (Table 1). Next, we attempted to isolate clones of the XR-FeLV provirus from ON-T and ON-C using PCR. Clone 10 from 293T/ON-T/#3 cells and clone 20 from 293T/ON-C/#9 cells were subsequently sequenced (Fig. 3). The results indicated that clones 10 and 20 contained the X-region approximately 841 and 689 bp from FcERV-gamma4 in their 5′-leader sequence, respectively (Fig. S6). However, the X-region did not have an open reading frame (ORF); therefore, both *gag* regions of the XR-FeLVs were disrupted (Fig. S7). Based on sequence analysis, we discovered that clone 10 from ON-T carried FeLV-A *env*, whereas clone 20 from ON-C carried FeLV-D *env* (Table 1; Fig. 3). The FeLV-A *env* sequence from clone 10 was similar to the FeLV-A *env* sequences observed in the ON family (Fig. 4). The FeLV-D *env* from clone 20 in ON-C belonged to clade D3 (Fig. 6). The previously isolated FeLV-D clones [FeLV-D/ON-T and FeLV-D re-T (19) from ON-T] did not contain the X-region. Our phylogenetic analysis indicated that the X-region of clone 10 was in close proximity to FcERV gamma4-X2, while the X-region of clone 20 formed a separate clade (Fig. 7). Our results suggest that it is unlikely that XR-FeLV was transmitted among the family of cats.

## DISCUSSION

FeLV recombinant viruses with endogenous retroviral sequences have occasionally been detected in FeLV-A-infected cats. In the present study, we first reported multiple recombination events involving endogenous retroviruses in at least two cats from the same family.

Thus far, four cases of FeLV-D infection have been confirmed by viral testing, and all of them were considered to be hematopoietic diseases (19); however, the pathogenicity of FeLV-D is not clear due to the small number of virus-positive cases. In this study, we were able to obtain FeLV-D-positive individuals and investigated the pathogenicity of FeLV-D. The presence of FeLV-D proviral clonality in ON-T, along with histopathological findings, suggests that FeLV-D was already present in the early stages of tumorigenesis and may be associated with tumor induction (Fig. 2). Only one FeLV-D proviral integration site was identified in ON-T, and it was not clear whether FeLV-D was integrated at several sites. After analyzing the viral integration site using the genome database, PPM1A, Six6, and DHRS7 were expected to be present in the vicinity of the FeLV-D integration site, and dysregulation of their genes by FeLV insertional mutagenesis may be associated with tumorigenesis. A comparison of FeLV-D viral loads at a specific integration site in the spleen, liver, and kidney tissues indicated similar viral loads in the spleen and liver but very low viral loads in the kidney. These contradictory histological results may be explained by the contamination of normal kidney tissue. Alternatively, the kidneys may have been infiltrated by other types of tumor cells although this was not confirmed. Sequence analysis of FeLV-D indicated that FeLV-D in ON-T was classified as Clade 1, while FeLV-D in ON-C was classified as Clade 1, 2, and 3. This suggests that FeLV-D in ON-T is of a single origin, while FeLV-D in ON-C may be derived from multiple origins. In ON-C, identical FeLV-D sequences were not observed between blood and cell line (AH927/ON-C/#9) samples. The reason for this is unknown although it could be due to the different materials and the fact that the viral gene sequences could not be determined in an exhaustive manner.

This study also identified four distinct strains of FeLV-B in ON-T, one of which was identical to that observed in ON-H, as determined by recombination site analysis. This demonstrates that FeLV-B may have been transmitted between ON-T and ON-H although the direction of virus transmission remains unknown. The most likely explanation is transmission from ON-H to ON-T, which is supported by the phylogenetic tree constructed using the enFeLV and FeLV-A regions (Fig. 5). However, as FeLV-B variation in the ON family cats was high, unobserved variants may provide alternative hypotheses regarding transmission.

The virus isolation results indicate that FeLV-B in ON-T was mediated by feline Pit1 and feline Pit2 as viral receptors, whereas the virus in ON-C was mediated by feline Pit1. This suggests that FeLV-B in ON-T and ON-C may belong to different strains of the same virus. Furthermore, a comparison of the FeLV-B sequences from ON-H and ON-T indicated that their viral receptor usage was similar, suggesting that transmission between ON-H and ON-T was the most likely scenario.

A previous study found XR-FeLV in 6.4% of FeLV-infected cats (44); however, its role in the disease is still being investigated. In this study, XR-FeLV was successfully isolated for the first time and was identified in ON-T and ON-C. The XR-FeLV proviruses (clone 10 and clone 20) isolated in this study had gene deletions in the *gag* and *pol* genes (Fig. 3; Fig. S7) and, therefore, are unlikely to be replication-competent. However, XR-FeLVs were successfully isolated from cultures, suggesting that XR-FeLV is thought to be replication-competent when in the presence of helper viruses. Moreover, FeLV-B and FeLV-D were only identified together with FeLV-A, which can be transmitted between individuals, suggesting that these viruses occurred *de novo* within an individual and have no horizontal transmission capacity.

Recombinant viruses with ERV are thought to affect the pathogenicity of FeLV-A infection through Env-pseudotyped viruses, thereby complicating the understanding of its pathogenesis. The frequency of the occurrence of recombinant viruses may be influenced by the expression of ERV or by the chance of encountering exRV and ERV. As ERV is thought to be silenced in normal tissues (45), it is possible that changes in the expression of ERV may occur in relation to the type of disease or disease progression. These changes may be associated with the frequency of occurrence of recombinant viruses, and the immune status of the host and the presence of resistance molecules may be associated with the emergence of recombinant viruses (46–49).

Because of the limitations associated with animal experiments using domestic cats, a detailed analysis of clinical cases in naturally infected cats will be necessary to clarify the pathogenicity of the recombinant virus with the ERV. In standard research methods, virus isolation is performed using susceptible cultured cells to characterize the viruses. However, it should be noted that in the course of this operation, there is the possibility of viral recombination and genetic mutation, as well as the possibility that viruses will not be isolated in culture cells. Thus, obtaining viral information from a variety of materials, including viral isolates, would be useful to rule out artificial sequences. Overall, there may be technical limitations.

In summary, we demonstrated that recombination between FeLV and ERVs can occur more frequently in nature than previously reported, suggesting that FeLV may be a good model to understand the effects of recombination events on viral pathogenicity. Understanding the multiple mechanisms underlying the emergence of these recombinant viruses will improve our understanding of FeLV infections. In addition, we believe that this study provides an opportunity to gain further insights into FeLV-mediated ERV-induced disease outbreaks and may shed light on the contribution of ERVs to pathogenic viruses.

## MATERIALS AND METHODS

### History of the domestic cat family

In the family consisting of cats with the prefix ON, a mother cat gave birth to four separate litters, as shown in Fig. 1. A female kitten was born in the first litter (ON-M), another female kitten was born in the second litter (ON-K), a male kitten was born in the third litter (ON-T), and a total of three kittens were born in the fourth litter [ON-H (female), ON-C (male), and ON-L (male)]. None of the cats received the FeLV vaccination and were allowed to roam freely inside or outside the house. Furthermore, all male cats were castrated.

### Sample processing

DNA was extracted from the blood, spleen, liver, and kidneys using a commercial kit (DNeasy Blood & Tissue Kit; QIAGEN, Hilden, Germany). The DNA concentration was determined using a NanoDrop 2000 spectrophotometer (Thermo Fisher Scientific, Waltham, MA, USA). Tissue samples were stored at −80°C, and DNA samples were stored at −20°C.

### FeLV and FIV status

Blood samples were tested for the FeLV gag antigen and FIV gag antibody using a commercially available test kit (SNAP FeLV/FIV combo kit; IDEXX Laboratories Inc., USA) in the Oishi Animal Clinic.

### Necropsy and histopathology

Clinical histories, necropsy results, and histopathological reports were retrieved from the individuals. Necropsy was performed in our laboratory. Thereafter, the samples were sent to IDEXX Laboratories (Tokyo, Japan) for histopathological examination.

### Cell lines

Fresh and infected human embryonic kidney (HEK293T) (50) and feline fibroblast (AH927) (51) cells were cultured in Dulbecco’s modified Eagle’s medium (DMEM) supplemented with 1 × penicillin/streptomycin and 10% fetal calf serum (FCS). GPLac cells (19), which is an *Env*-negative packaging cell line containing murine leukemia virus (MuLV) *gag-pol* gene and a-galactosidase (LacZ)-encoding pMXs retroviral vector, and 293Lac cells (20) containing a LacZ-encoding pMXs retroviral vector were cultured in DMEM supplemented with 1 × penicillin/streptomycin and 10% FCS. AH927Lac cells were established by infecting AH927 cells with a LacZ-coding retroviral vector using a pseudotyped FeLV-B virus (9). The LacZ-coding retroviral vector was rescued in the presence of replication-competent viruses in HEK293T and AH927Lac cells. AH927Lac cells were maintained in DMEM supplemented with 1 × penicillin/streptomycin, 10% FCS, and 2 µg/mL Puromycin (Nacalai Tesque, Kyoto Japan). *Mus dunni* tail fibroblast (MDTF) cells (52), MDTF cells expressing feline Pit1(MDTF-fePit1), and MDTF cells expressing feline Pit2 (MDTF-fePit2) (20) were cultured in the same medium mentioned above. All cells were cultured in a humidified atmosphere at 37°C with 5% CO_2_ and were passaged every 3–4 days once they reached 70%–80% confluence.

### Viral isolation

To isolate FeLV from the infected cats, different sampling sources were prepared to infect the target cells. Cell lines were generated using HEK293T and AH927 cells to isolate FeLV from the ON-T and ON-C cats. The samples, including the spleen, PBMCs, and plasma, from either ON-T or ON-C, are described in Table 1. Infected cell lines were subjected to further analyses.

### Quantitative PCR of FeLV-D provirus

Quantitative real-time PCR was conducted using SYBR Premix Ex Taq II (Tli RNase H Plus; Takara) on a CFX96 Touch Real-Time PCR Detection System (Bio-Rad, Hercules, CA, USA). The 5′ flanking region of the FeLV-D/ON-T provirus (34) was amplified using primers Fe-702S (5′-ACCTGCACTCCTCAGCTTAC-3′) and Fe-713R (5′-CATCGGCTTTCTGCCATGTG-3′). The region between the 5′ flanking region and 5′LTR of the FeLV-D/ON-T provirus was amplified using the primers Fe-703S (5′-ACCACATGGCAGAAAGCCGATGG-3′) and Fe-714R (5′-TAAGGTGGGGGGTTTGAGCCA-3′). The copy number was calculated using the FeLV-D/ON-T plasmid (34). The proviral load was calculated using the following formula: Proviral load (%) = [Copy number of “5'flanking - 5'LTR”/(Copy number of “5'flanking internal”/2)] × 100.

### Pseudotyped viruses

To prepare Env-pseudotyped viruses carrying LacZ, GPLac cells were transfected with the env expression plasmids of FeLV-A (FeLV clone33), FeLV-B (Gardner-Arnstein), FeLV-C (Sarma), FeLV-D (ON-T), and FeLV-E (FeLV-TG35-2) in six-well plates (20). After 72 h, the culture supernatants were filtered through a 0.22-µm filter and stored at −80°C until use. Transfection of env expression plasmids was performed using the TransIT-293 transfection reagent (Takara, Shiga, Japan) according to the manufacturer’s instructions.

### Viral interference assay

HEK293T, HEK293T/ON-T/#0, HEK293T/ON-T/#3, AH927, and AH927/ON-C/#9 cells were seeded in 24-well plates at a concentration of 1–3 × 10^4^ cells per well, one day prior to infection. Each cell line was then incubated separately with 250 µL of every virus listed above, along with polybrene (10 µg/mL). After 48 h of incubation, the cells were stained with 5-bromo-4-chloro-3-indolyl-β-d-galactopyranoside (X-Gal), and single-cycle infectivity was evaluated by counting the blue-stained nuclei under a microscope. Viral titers were calculated as the log of infectious units per milliliter, along with standard deviations.

### Infection assay

To determine the receptor usage of the FeLV-B strains, supernatants from 293Lac cells infected with the supernatant of 293T/ON-T #3 cells, and AH927 Lac cells infected with the supernatant of AH927/ON-C #9 cells, were prepared for the virus infection assay. Target cells, including HEK293T, AH927, MDTF, MDTF-fePit1, and MDTF-fePit2 cells, were seeded at a concentration of 1–3 × 10^4^ cells per well in 24-well plates, 1 day prior to infection. Each cell line was then incubated separately with 250 µL of each virus and supplemented with polybrene (10 µg/mL). After 48 h of incubation, the cells were stained with X-Gal and single-cycle infectivity was evaluated by counting the blue-stained nuclei visualized under a microscope. Viral titers were calculated as the log of infectious units per milliliter, along with standard deviations.

### PCR and sequencing

PCR amplification was conducted using the KOD One PCR master mix (TOYOBO Bio Inc., Osaka, Japan), according to the manufacturer’s instructions, with DNA extracted from cultured cells, tissues, and blood using the DNeasy Blood and Tissue Kit (Qiagen, Osaka, Japan). To identify different subgroups of FeLV using PCR, specific primer pairs were used to amplify the target regions of FeLV-D, FeLV-B, and XR-FeLV in the sample. The primer pairs used were Fe-14S, Fe-3R, Fe-917S (5′-ATGAAACCCCCAGCGGGAATGG-3′)/918S (5′-AAGGGTAGACATGGGAATTGGAGC-3′), and Fe-7R for FeLV-D (19), PRB1 and Fe-3R for FeLV-B (9), and Fe-23S and Fe-110R for XR-FeLV (44). To further investigate the FeLV subgroups, the full-length *env* region of FeLV was amplified using primer pairs Fe-4S/Fe-9S and Fe-3R/Fe-7R (9). The amplified fragment was then cloned into the pCR4-Blunt Topo vector (Invitrogen, Waltham, MA, USA). Several clones from each subgroup were selected and sequenced to obtain genetic information, confirming the presence of various FeLV subgroups and XR-FeLV in the sample DNA. The primer sequences used for cloning are shown in Fig. S8.

### Cloning of full-length FeLV

The full-length FeLV was amplified by PCR using the Fe-23S and Fe-7R primer pair located at positions 8307–8328 and positions 8336–8357 of the 3′LTR of FeLV-A/clone33 (Accession no. AB060732.2). The amplified product was subsequently cloned into the pCR4-Blunt Topo vector (Invitrogen, Waltham, MA, USA) and sequenced.

### Phylogenetic analysis of FeLV-A env and XR-FeLV

The reference sequences were obtained from GenBank and are listed in Fig. S9. Alignment was performed using MUSCLE software (53). Two different software packages were used for phylogenetic analysis. The most suitable method for phylogenetic analysis at the nucleic acid level was determined using the jModel test (54). MEGA X was used for phylogenetic analysis (55). A phylogenetic tree was constructed using the Maximum Likelihood (ML) method, and the General Time Reversible model with the lowest Bayesian Information Criterion (BIC) score (56). A discrete Gamma distribution [with five categories (+G, parameter = 0.6714)] was selected to model evolutionary rate differences among sites. Bootstrap values from 1,000 replicates were used as branch junction percentages to assess nodal support.

### Genetic analyses of FeLV-B and FeLV-D

Two analyses were performed to investigate the possibility of FeLV-B and FeLV-D transmission within the household. First, concordance or discordance was investigated in the recombination junctions of the FeLV-B and FeLV-D clones identified from the ON family cats. Second, phylogenetic analysis was performed using the enFeLV and FeLV-A regions from FeLV-B, and the ERV-DC region from FeLV-D. Multiple sequence alignments (MSAs) were constructed by MAFFT (version 7.508) (57) using the *env* sequences of the 12 FeLV-B, and 16 FeLV-D isolates identified in the ON family cats and reference sequences (Table 1; Fig. S9). FeLV-B and FeLV-D recombinant junctions were identified using Simplot++ (version 1.3) (58). For FeLV-B phylogenetic analysis, partial MSAs were extracted from the recombinant junctions: (i) sites 1–1192 (enFeLV derived region, Fig. 5B) and (ii) sites 1619–2550 (FeLV-A derived region, Fig. 5A). Phylogenetic trees were constructed using the ML method in IQ-TREE (version 2.1.4) (59). Substitution models were selected based on the BIC score provided by ModelFinder (60): TN + F for sites 1–1192 (enFeLV derived region) and HKY + F for sites 1619–2550 (FeLV-A derived region). Branch supports were measured as ultrafast bootstrap values given by UFBoot2 (61) with 1,000 replicates. Similarly, for the FeLV-D phylogenetic analysis, partial MSAs were extracted from the recombinant junction site 48–1560 (ERV-DC region, Fig. 6A). Phylogenetic tree was constructed using the ML method and branch supports were evaluated by bootstrapping (1,000 times). The substitution models were selected based on the lowest BIC score, K2+G for site 48–1560 (ERV-DC derived region) (62). The MEGAX (version 10.1) software package was used for the phylogenetic analysis (55).

### Statistical analysis

The results of the FeLV-D interference assay were considered statistically significant if *P*-values were <0.05, as determined using the Student’s *t*-test and one-way analysis of variance.

## Data Availability

The nucleotide sequences reported in this study were deposited in the DDBJ, EMBL, and GenBank databases under accession numbers LC765227–LC765258. The data, materials, and codes for FeLV-B genetic analysis are available at https://github.com/Junna-Kawasaki/FeLV-B_2023.

## SUPPLEMENTAL MATERIAL

Supplemental figures jvi.01400-23-s0001.pdf Figures S1 to S9.

ASM does not own the copyrights to Supplemental Material that may be linked to, or accessed through, an article. The authors have granted ASM a non-exclusive, world-wide license to publish the Supplemental Material files. Please contact the corresponding author directly for reuse.
