# Supplementary material for: Multiple recombination events between endogenous retroviral elements and feline leukemia virus
Source: J Virol. 2024 Jan 19;98(2):e01400-23. doi: 10.1128/jvi.01400-23 (PMC10878261; doi:10.1128/jvi.01400-23)
Supplement: Supplemental figures — Figures S1 to S9. [file jvi.01400-23-s0001.pdf]

Supplemental Information

Supplemental Figure Legends

Figure S1. Results of the PCR analysis of FeLV-D (A) and XR-FeLV (B) using DNA samples from the blood of ON-C, ON-L, ON-H, and ON-T. The control is a DNA sample from the blood of a FeLV-negative cat.

Figure S2. Necropsy and histopathology of a cat (ON-T) infected with FeLV. (A) Conjunctival pallor in the eyes; (B) mucosal pallor in the gums; (C) the mass within the thoracic cavity is indicated by a dotted line; (D) a mass was observed in a portion of the base of the heart. Histopathological lesions in organs of the FeLV-infected cat are shown as low-magnification (E–H) and high-magnification (I–L) images. Black scale bar: 200 µm in (E–H) and 50 µm in (I–L). (E, I) Lesions in the heart; (E) the black arrows indicate lymphoid cell invasion, (I) the yellow arrow indicates an atypical erythrocyte, and the black arrows indicate macrophages; (F, J) lesions in the liver; (F) the black arrow indicates the central vein with sporadic round cell clusters between hepatocytes, (J) the green squares indicate normal hepatocytes, the red arrows indicate atypical erythroblasts, and the black arrows indicate the proliferation of atypical micromegakaryocytes; (G, K) lesions in the kidney; (G) the black arrow indicates neoplastic infiltration, (K) the black arrows indicate atypical megakaryocytes, and the red arrows indicate atypical erythroblasts; (H, L) lesions in the spleen; (H) the black arrow indicates neoplastic infiltration, and (L) the black arrows indicate atypical micromegakaryocytes.

Figure S3. SimPlot++ analysis of the recombination junction of FeLV-B identified from ON family cats. The analysis was performed using Simplot++ software version

1.3, with a window width of 200 bp and a step size of 20 bp. The query sequence was compared with each of the reference strains at each position of the window. The nucleotide position of the analyzed genome region is displayed on the x-axis, and the percentage of nucleotide identities between the query sequence and the reference strain is shown on the y-axis. The lines indicate enFeLV (blue), FeLV-A (orange), and ERV-DC10 (purple). Recombination site is indicated by a dotted line.

Figure S4. Amino acid alignment of envelope variable region A of FeLV-B and enFeLV. Amino acid residues are shown in single-letter code, and conserved residues are indicated with a dot. The amino acid position 73 was previously reported to be associated with the receptor usage of FeLV-B (23).

Figure S5. Recombination junction of FeLV-D identified from ON family cats. The analysis was performed using Simplot++ software version 1.3, with a window width of 200 bp and a step size of 20 bp. The query sequence was compared to each of the reference strains at each position of the window. The nucleotide position of the analyzed genome region is displayed on the x-axis, and the percentage of nucleotide identities between the query sequence and the reference strain is shown on the y-axis. The lines indicate enFeLV (pink), FeLV-A (dark brown), and ERV-DC8 (dark blue). Recombination site is indicated by a dotted line.

Figure S6. Multiple sequence alignment of nucleic acid sequences of the X-region. The X-region of XR-FeLV (FeLV-A_ON-T_Provirus_clone_10) and XR-FeLV (FeLV-D_ON-C_Provirus_clone_20) were aligned with FcERV-gamma4-X2. Nucleic acid sequences are shown as single-letter codes, and conserved nucleotides are indicated with a dot. (-) indicates no sequence alignment.

Figure S7. Schematic structures of the X-region. Schematics of the structures from the 5′ long terminal repeat (LTR) U3, 5′ -leader, and *gag* gene of XR-FeLV (FeLV-A_ON-T_Provirus_clone_10) and XR-FeLV (FeLV-D_ON-C_Provirus_clone_20). The prototypic structures of the FeLV *gag* gene and its protein-coding domains are indicated on the top with reference to the FeLV-A clone 33 strain (sp, signal peptide; MA, matrix protein; p12, p12 protein; CA, capsid protein; NC, nucleocapsid protein). The structure of the 5′LTR, 5′ -leader, and *gag* gene of FcERV gamma 4-X2 is indicated on the bottom. This schematic focuses on the insertion of the X region (black boxes). An origin of the X-region is also marked in gray on FeERV-Gamma4-X2.

Figure S8. Primer pairs used for the cloning of FeLV. PCR primers used for the PCR-mediated amplification of viral genes are shown on the FeLV provirus schematic. Schematic of the FeLV proviral genome highlighting the functional features: the 5′ long terminal repeat (LTR), *gag*, *pol*, *env*, and the 3′ LTR.

Figure S9. **Reference sequences used in this study.**

Figure S1

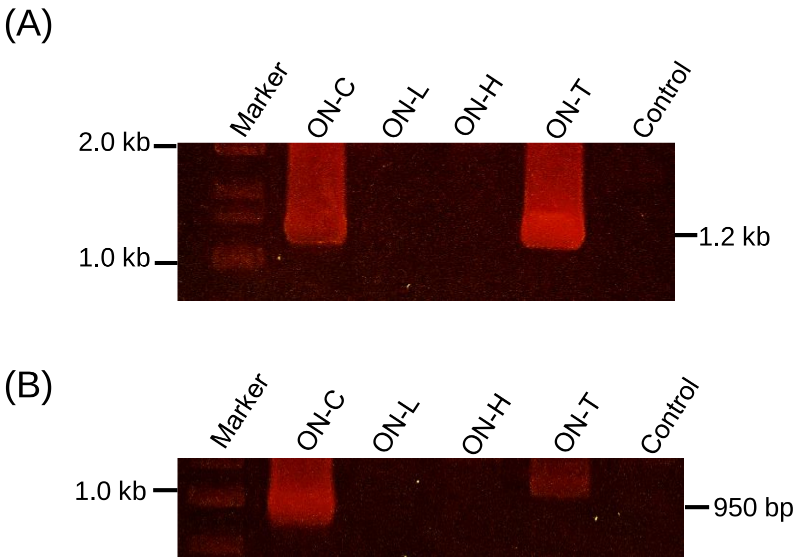
(A)
Marker
ON-C
ON-L
ON-H
ON-T
Control
2.0 kb
1.0 kb
1.2 kb
(B)
Marker
ON-C
ON-L
ON-H
ON-T
Control
1.0 kb
950 bp

Figure S2

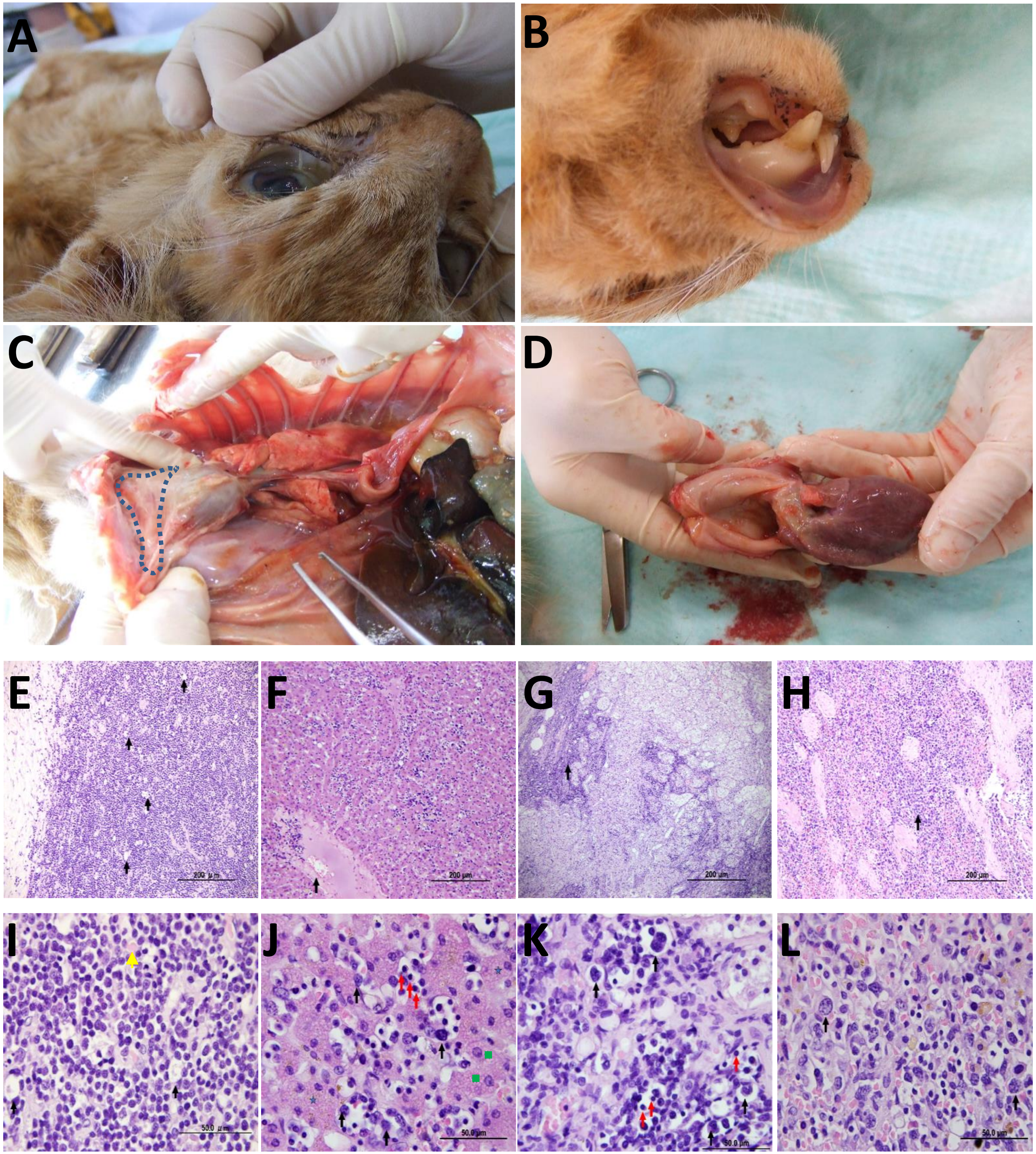

Figure S3

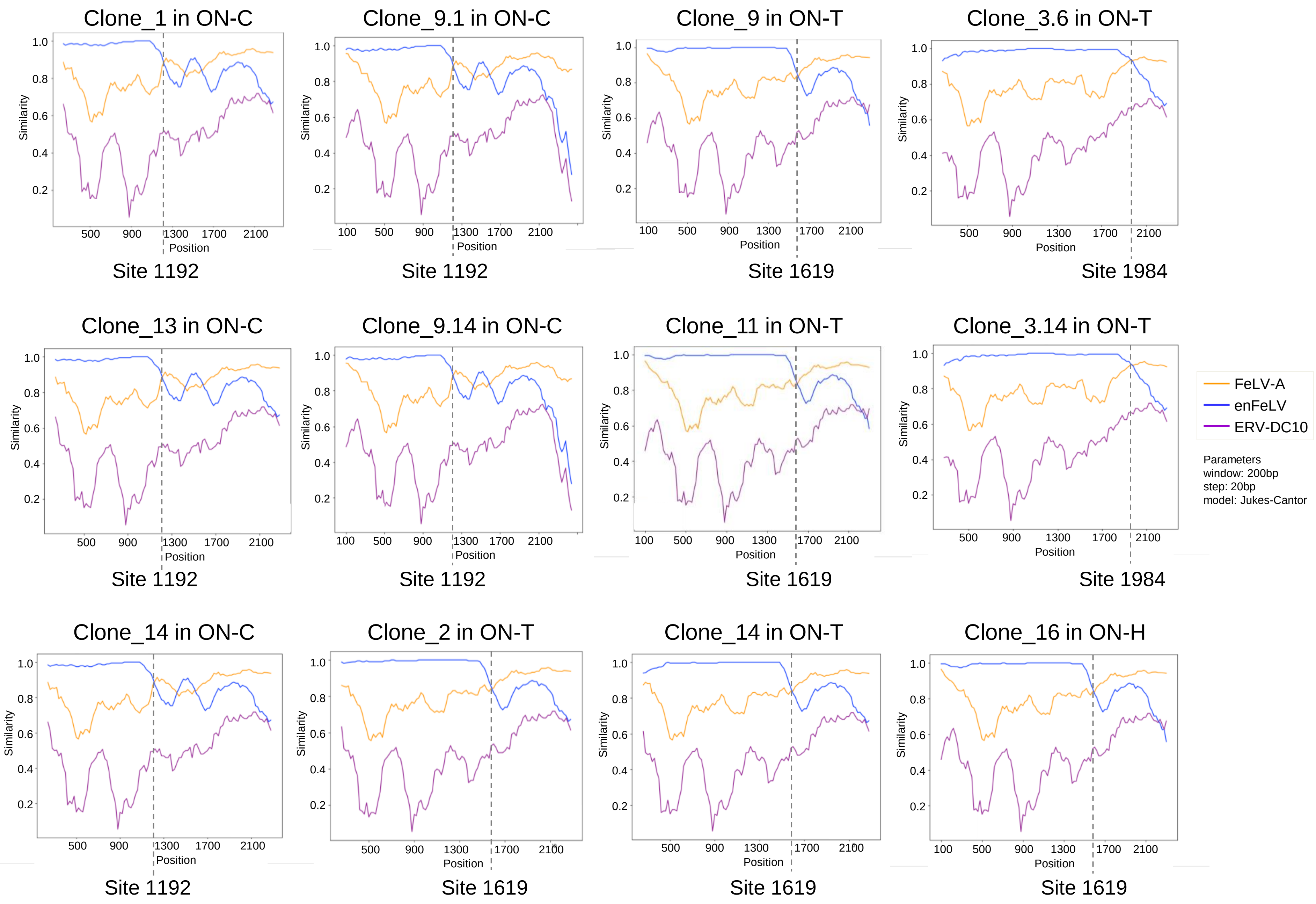
Clone_1 in ON-C
Site 1192
Clone_9.1 in ON-C
Site 1192
Clone_9 in ON-T
Site 1619
Clone_3.6 in ON-T
Site 1984
Clone_13 in ON-C
Site 1192
Clone_9.14 in ON-C
Site 1192
Clone_11 in ON-T
Site 1619
Clone_3.14 in ON-T
Site 1984
Clone_14 in ON-C
Site 1192
Clone_2 in ON-T
Site 1619
Clone_14 in ON-T
Site 1619
Clone_16 in ON-H
Site 1619
Similarity
Position
FeLV-A
enFeLV
ERV-DC10
Parameters
window: 200bp
step: 20bp
model: Jukes-Cantor

Figure S4

Variable Region A (VRA): positions 45 – 73 – 91 (position 73 boxed)

Sequence	45 → 91
enFeLV-AGTT	C D I I G N T W N P S D Q E P F P G Y G C D Q P M R R W Q Q R N T P F Y V C P G H A N R K Q C
FeLV-B (Gardner-Arnstein)	. . . .
FeLV-B ON-T clone 9	. . . . R . . . .
FeLV-B ON-T clone 11	. . . . R . . . .
FeLV-B ON-T clone 3.14	. . . V A . . . . R . . . .
FeLV-B ON-T clone 3.6	. . . V A . . . . R . . . .
FeLV-B ON-T clone 14	. . . . R . . . .
FeLV-B ON-T clone 2	. . . . . S . . . . R . . . .
FeLV-B ON-H clone 16	. . . . R . . . .
FeLV-B ON-C clone 9.14	. . . . R . . . .
FeLV-B ON-C clone 9.1	. . . . R . . . .
FeLV-B ON-C clone 13	. . . . R . . . .
FeLV-B ON-C clone 1	. . . . R . . . .
FeLV-B ON-C clone 14	. . . . R . . . .

Figure S5

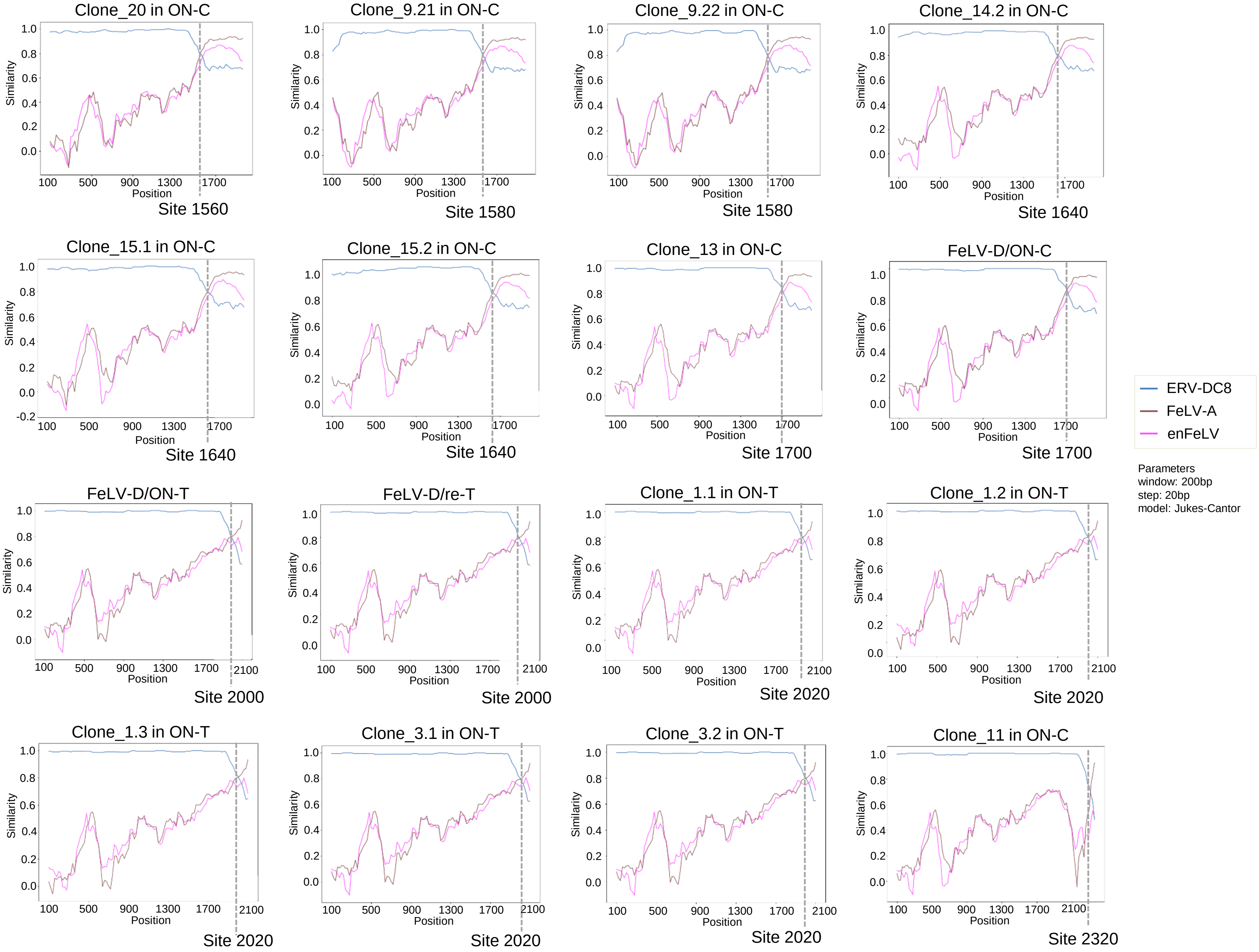

Clone_20 in ON-C
Site 1560
Clone_9.21 in ON-C
Site 1580
Clone_9.22 in ON-C
Site 1580
Clone_14.2 in ON-C
Site 1640
Clone_15.1 in ON-C
Site 1640
Clone_15.2 in ON-C
Site 1640
Clone_13 in ON-C
Site 1700
FeLV-D/ON-C
Site 1700
FeLV-D/ON-T
Site 2000
FeLV-D/re-T
Site 2000
Clone_1.1 in ON-T
Site 2020
Clone_1.2 in ON-T
Site 2020
Clone_1.3 in ON-T
Site 2020
Clone_3.1 in ON-T
Site 2020
Clone_3.2 in ON-T
Site 2020
Clone_11 in ON-C
Site 2320
Similarity
Position
ERV-DC8
FeLV-A
enFeLV
Parameters
window: 200bp
step: 20bp
model: Jukes-Cantor

Figure S6

```
FcERV gamma4-X2                          1   TGGGGGCCTTCTTGGCCCGTCTGGGGACCTTCTTGGTCCATCTGGGGACCTTCTTGGTCCGTCTGGGGGCCTTCTTGGCC 80
XR-FeLV FeLV-A_ON-T_provirus_clone_10    1   ..................A....................G........................................ 80
XR-FeLV FeLV-D_ON-C_provirus_clone_20        --------------------------------------------------------------------------------

FcERV gamma4-X2                          81  CGTCTGGGGACCTTCTTGGTCCATCTGGGGGCCTTCTTGGCCCATCTGGGGACCTTCTTGGTCCATCTGGGGACCTTCTT 160
XR-FeLV FeLV-A_ON-T_provirus_clone_10    81  .........G.........C..G.......A.........T..........G.........C.................. 160
XR-FeLV FeLV-D_ON-C_provirus_clone_20        --------------------------------------------------------------------------------

FcERV gamma4-X2                          161 GGTCCATCGGGGGCCTTTTTTGGCCCATCGGGATTCTTTTCTGTGGGCTGCTTACGCCCAACGCGCCTGCGAACTAAATA 240
XR-FeLV FeLV-A_ON-T_provirus_clone_10    161 ............................T................................................... 240
XR-FeLV FeLV-D_ON-C_provirus_clone_20    1   ---------------------------------............................................... 47

FcERV gamma4-X2                          241 CTTTCATTTTCTCTATGAACTTCCAGAGTGCTAAGAAATATCTCTAGGCGTCTAAAAG------AAAAAACCATCTAGGC 314
XR-FeLV FeLV-A_ON-T_provirus_clone_10    241 ..........................................................------................ 314
XR-FeLV FeLV-D_ON-C_provirus_clone_20    48  .............................A............................AAAAAA................ 127

FcERV gamma4-X2                          315 ATGCCAATTGTTATCCGTATTTTGATGTGATGTGTGTCTGTGTGTCTGTCTGTTAAATCGACTGGAATGATTGTTGGGAG 394
XR-FeLV FeLV-A_ON-T_provirus_clone_10    315 ................................................................................ 394
XR-FeLV FeLV-D_ON-C_provirus_clone_20    128 ...........C.................................................................... 207

FcERV gamma4-X2                          395 TCAGGGGCACGCGCCTGACTTCCCCTATGTGTAGTCCCACAGCATAAGCTACGGGATTCGAGTGGGCTCTAACCCGACTT 474
XR-FeLV FeLV-A_ON-T_provirus_clone_10    395 ................................................................................ 474
XR-FeLV FeLV-D_ON-C_provirus_clone_20    208 ..............................................C................................. 287

FcERV gamma4-X2                          475 CCGCGGTGATCCTCATACGGCTTAAGGCGGTCAAAATTTATTTTGATCCAAGCAGGGGGTTTATACCTGCCCGCCCATGC 554
XR-FeLV FeLV-A_ON-T_provirus_clone_10    475 ................................................................................ 554
XR-FeLV FeLV-D_ON-C_provirus_clone_20    288 ...........................................................................T.... 367

FcERV gamma4-X2                          555 TAAGAGGCACCTAAGTTCCCGCGAGGGACGCGGACGGGCGAGTACGCGAGTGCTTCTATGTCAGTCTAAATGTACTGTCA 634
XR-FeLV FeLV-A_ON-T_provirus_clone_10    555 ..........................................................................T..... 634
XR-FeLV FeLV-D_ON-C_provirus_clone_20    368 ..........................................................................T..... 447

FcERV gamma4-X2                          635 CCCCTGGGCCCTTGTAATTACTGCCGTCCTAGCCATAACCCTTTCTGTTCGGCTGGCCGAG-----ACCAGAAAGTTGGA 709
XR-FeLV FeLV-A_ON-T_provirus_clone_10    635 ..............................................................-----............. 709
XR-FeLV FeLV-D_ON-C_provirus_clone_20    448 ...................G.C...........................G............ACGGC............. 527

FcERV gamma4-X2                          710 ACCGCTGCGAAAGATTCTTGTTAGTTCTTGTTTTTCTTTCATGGGTCGGATGTTTTATTGGAATTAAGTGTTTTATCGGC 789
XR-FeLV FeLV-A_ON-T_provirus_clone_10    710 ................................................................................ 789
XR-FeLV FeLV-D_ON-C_provirus_clone_20    528 ................................................................................ 607

FcERV gamma4-X2                          790 TGATCAGAATTAATTATTCCATCCTTTGTTCCTCTCTCCTCCTTCCTCAGCTCCCTGCGGCTTTCCCTCCCACCCCCTGG 869
XR-FeLV FeLV-A_ON-T_provirus_clone_10    790 ....................................................---------------------------- 841
XR-FeLV FeLV-D_ON-C_provirus_clone_20    608 ...........................................................................T.... 687

FcERV gamma4-X2                          870 CC 871
XR-FeLV FeLV-A_ON-T_provirus_clone_10    841 -- 841
XR-FeLV FeLV-D_ON-C_provirus_clone_20    688 .. 689
```

Figure S7

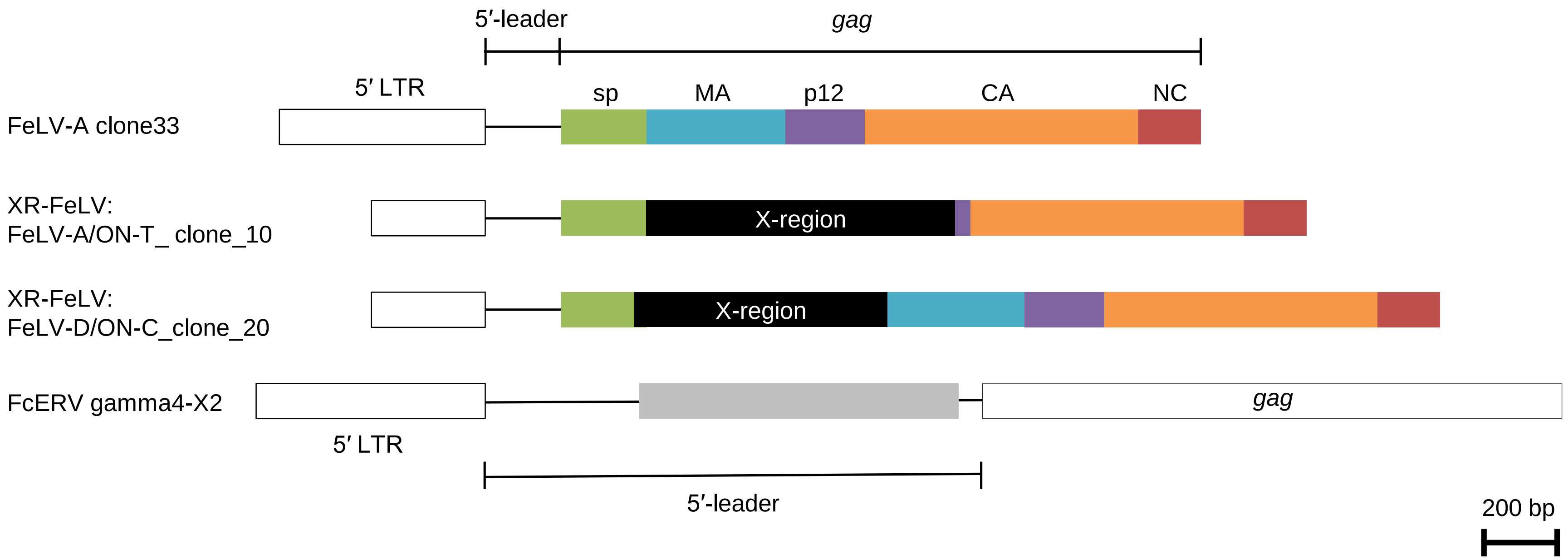

5′-leader
gag
5′ LTR
sp
MA
p12
CA
NC
FeLV-A clone33
XR-FeLV:
FeLV-A/ON-T_ clone_10
X-region
XR-FeLV:
FeLV-D/ON-C_clone_20
X-region
FcERV gamma4-X2
gag
5′ LTR
5′-leader
200 bp

Figure S8

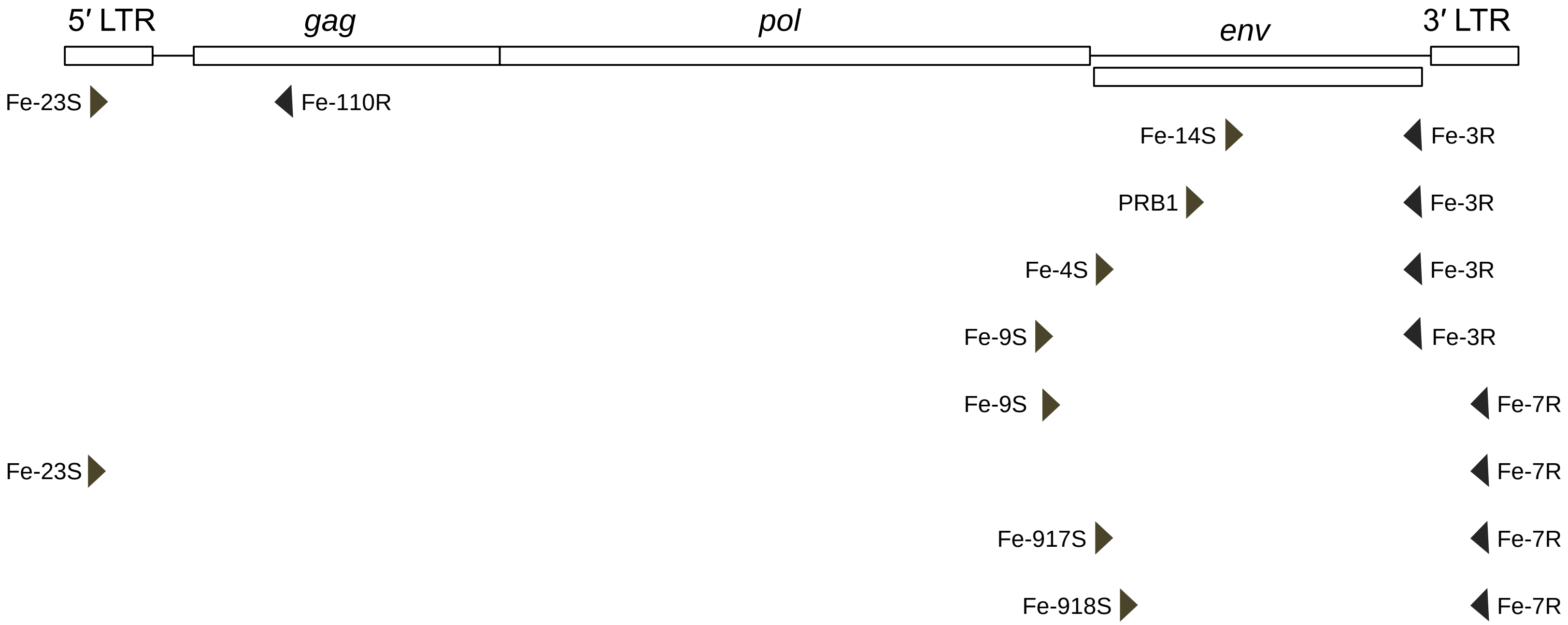

5′ LTR
gag
pol
env
3′ LTR
Fe-23S
Fe-110R
Fe-14S
Fe-3R
PRB1
Fe-3R
Fe-4S
Fe-3R
Fe-9S
Fe-3R
Fe-9S
Fe-7R
Fe-23S
Fe-7R
Fe-917S
Fe-7R
Fe-918S
Fe-7R

Figure S9

Reference sequences used in this study

Description	Accession no.
FeLV-A (61E)	M18247
FeLV-A (Clone 33)	AB060732
FeLV-A (Glasgow-1)	KP728112
FeLV-A (GF37-1)	AB635849
FeLV-A (IS20-1NF)	AB635537
FeLV-A (KG20-2)	AB635639
FeLV-A (KM7-1)	AB635707
FeLV-A (ME29-1)	AB635554
FeLV-A (MG3-1)	AB635495
FeLV-A (O27ThyT-1)	AY706357
FeLV-A (ON33-1)	AB635483
FeLV-A (TY5-1)	AB635500
FeLV-A (TY26-2)	AB635514
FeLV-A (YG13-1)	AB635545
FeLV-B (Gardner-Arnstein)	X00188
FeLV-D (re-T)	AB673432
FeLV-D (ON-T)	AB673426
FeLV-D (ON-C)	AB673429
enFeLV (GGAG)	AY364319
enFeLV (AGTT)	AY364318
ERV-DC (ERV-DC8)	LC597234
ERV-DC (ERVDC10)	AB674444
XR-FeLV (g48-2)	AB898757
XR-FeLV (g48-3)	AB898758
XR-FeLV (gKM13-5)	AB898766
XR-FeLV (5603-2)	LC176811
XR-FeLV (3532-2)	LC176812
XR-FeLV (g1370-4)	AB898761
XR-FeLV (g1370-6)	AB898762
XR-FeLV (gIK10-5)	AB898763
XR-FeLV (2610-4)	LC176813
FcERV (gamma 4-X2)	LC176799
FcERV (gamm 4-B4)	LC176798
FcERV (gamma 4-A1)	LC176795
FcERV (gamma 4-X)	LC176794
FcERV (gamma 4-3)	LC176792
FcERV (gamma 4-C1)	LC176796
FcERV (gamma 4-B3)	LC176793
FcERV (gamma4 4-1)	LC176791
FcERV (gamm 4-E2)	LC176797
